# Functionalized Spiral‐Rolling Millirobot for Upstream Swimming in Blood Vessel

**DOI:** 10.1002/advs.202200342

**Published:** 2022-03-31

**Authors:** Liu Yang, Tieshan Zhang, Rong Tan, Xiong Yang, Dong Guo, Yu Feng, Hao Ren, Yifeng Tang, Wanfeng Shang, Yajing Shen

**Affiliations:** ^1^ Department of Biomedical Engineering City University of Hong Kong Tat Chee Avenue Kowloon Hong Kong China; ^2^ CAS Key Laboratory of Human‐Machine Intelligence‐Synergy Systems Shenzhen Institutes of Advanced Technology Chinese Academy of Sciences Shenzhen 518057 China; ^3^ Guangdong Provincial Key Laboratory of Robotics and Intelligent System Shenzhen Institutes of Advanced Technology Chinese Academy of Sciences Shenzhen 518057 China; ^4^ Shenzhen Research Institute City University of Hong Kong Shenzhen 518057 China

**Keywords:** magnetic control, spiral‐rolling, untethered millirobots, upstream swimming

## Abstract

Untethered small robots with multiple functions show considerable potential as next‐generation catheter‐free systems for biomedical applications. However, owing to dynamic blood flow, even effective upstream swimming in blood vessels remains a challenge for the robot, let alone performing medical tasks. This paper presents an untethered millirobot with a streamlined shape that integrates the engine, delivery, and biopsy modules. Based on the proposed spiral‐rolling strategy, this robot can move upstream at a record‐breaking speed of ≈14 mm s^−1^ against a blood phantom flow of 136 mm s^−1^. Moreover, benefiting from the bioinspired self‐sealing orifice and easy‐open auto‐closed biopsy needle sheath, this robot facilitates several biomedical tasks in blood vessels, such as in vivo drug delivery, tissue and liquid biopsy, and cell transportation in rabbit arteries. This study will benefit the development of wireless millirobots for controllable, minimally invasive, highly integrated, and multifunctional endovascular interventions and will inspire new designs of miniature devices for biomedical applications.

## Graphical abstract

The ability to move against blood flow is essential for untethered small‐scale robots in blood vessels. The developed millirobot with a streamlined shape can swim at ≈14 mm s^−1^ against a flow of 136 mm s^−1^ through spiral‐rolling. Besides, in vivo drug delivery, tissue and liquid biopsy, and cell transportation are demonstrated in rabbit arteries via cavity and biopsy needle.

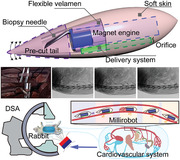

## 1 Introduction

Untethered miniature devices that can access narrow and complex regions inside the body not only provide promising solutions for minimally invasive diagnosis and treatment, but also help physicians perform operations away from the X‐ray source and potentially avoid their risk of radiation exposure and occupational diseases.^[^ ^1^, ^2^, ^3^, ^4^, ^5^, ^6^, ^7^, ^8^ ^]^ Moreover, sending the guidewire to the target is still a relatively time‐consuming and challenging task in current interventional surgery; therefore, the physicians’ experience may influence the clinical outcomes. This can be alleviated by untethered millirobots with wireless controllability and auto‐navigation ability to provide standard and automatic operations, which help physicians eliminate repetitive and complicated operations and focus on intraoperative decision‐making and risk management.

Recent progress in robotics has introduced several concepts for designing untethered miniature devices. Accordingly, several prototypes have been demonstrated, such as the microrobots for cell transplantation,^[^ ^9^, ^10^ ^]^ drug delivery in the stomach,^[^ ^11^ ^]^ prolonged drug retention in intestines,^[^ ^12^ ^]^ enhanced local hyperthermia,^[^ ^13^, ^14^ ^]^ precision surgery,^[^ ^15^, ^16^ ^]^ and biopsy in the gastrointestinal tract.^[^ ^17^, ^18^ ^]^ Despite the above achievements, driving and controlling medical robots inside the body remains significant challenges because of the complex in vivo environment,^[^ ^19^ ^]^ particularly inside blood vessels with blood flow resistance. Some proofs‐of‐concept have been proposed to propel against the flow by locomotion on or close to the channel wall,^[^ ^20^ ^]^ such as magnetically driven Janus microrollers (propelled against physiologically relevant blood flow up to 2.5 dyn cm^−2^) and an acousto‐magnetic controlled microswarm (upstream motion against the stream as high as 1.2 mm s^–1^).^[^ ^21^, ^22^ ^]^ However, realizing the controllable locomotion of these robots inside blood vessels is still challenging, let along conducting in vivo medical tasks, such as drug delivery and biopsy.^[^ ^23^, ^24^, ^25^, ^26^, ^27^ ^]^

**Figure 1:**
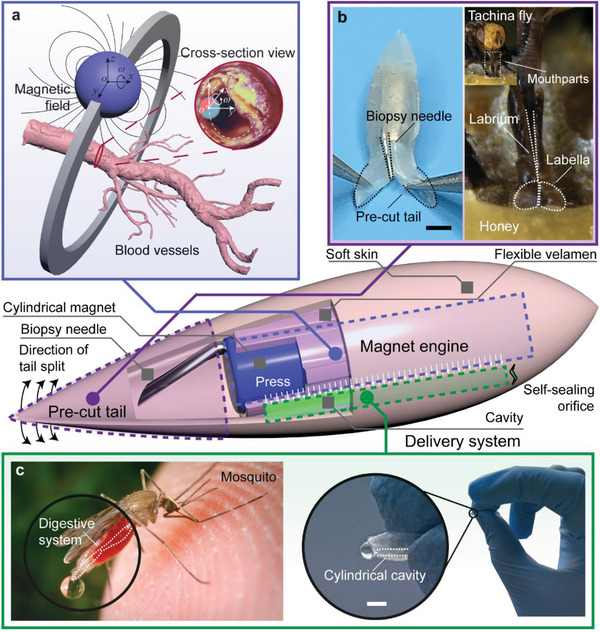
Schematics of the millirobot. The robot is designed to have a streamlined shape with three modules, that is, magnet engine, biopsy module, and delivery system. A cylindrical magnet covered by the flexible velamen is placed at the center of the robot to serve as the engine. A fine needle is attached to the magnet for endovascular biopsy and is concealed by a pre‐cut tail, which can be controllably opened and closed. Between the soft skin (outer layer) and the flexible velamen (inner layer), a cylindrical hole with a self‐sealing orifice is made for delivery. a) Overview of the magnetically controlled robotic system. A permanent magnet outside the blood vessel simultaneously rotates about the center of the vessel as well as its own center. b) Pre‐cut tail developed to have such a structure to expose and conceal the biopsy needle easily, inspired by the mouthparts of the tachina fly. Scale bar: 1.5 mm. Tachina fly reproduced under the creative commons license CC BY‐SA 2.5. Copyright Richard Bartz.  c) Designed pressure‐based delivery system, where the cavity will squeeze out the materials inside itself, inspired by the mosquito's digestive system. The cylindrical magnet will press the cavity for controlled release driven by a strong magnetic field. Scale bar: 2 mm. Mosquito reproduced under the creative commons license CC0. Copyright James Gathany by PIXNIO.

Here, we present an untethered millirobot with a streamlined shape (**Figure** **1**; Video S1, Supporting Information), whose biocompatibility and safety were confirmed by the cell viability test on L929 cells and the histological results of rabbit abdominal arteries. Remotely driven by the magnetic field (magnetic flux density ≈ 12 mT, magnetic field gradient ≈ 45 Gs cm^−1^), our robot can reach a forward speed of ≈14 mm s^−1^ against a blood phantom flow as high as ≈136 mm s^−1^ (mean flow rate ≈ 2680 mm^3^ s^−1^ in a tube with an inner diameter of 5 mm) through on‐wall spiral rolling. Moreover, inspired by the mosquito and fly in nature, we designed a pressure‐based delivery module with a self‐sealing orifice and (easy‐to‐open and auto‐closed tail) biopsy module for the robot and demonstrated drug delivery, tissue (thrombosis and endothelium) biopsy, liquid (blood) biopsy and cell (red blood cells) transportation in vivo in rabbit abdominal arteries (Video S2, Supporting Information), which were also driven and controlled by a magnetic field (magnetic flux density 12–220 mT, magnetic field gradient 45–550 Gs cm^−1^). This study demonstrates the concept of using milli‐robots in blood vessels and will inspire new designs of biomedical tools and benefit biomedical applications.

## 2 Results

### 2.1 Millirobot Design

The main body of the robot was fabricated by microcasting with soft silicones (Figure S1, Supporting Information), and its biocompatibility was verified via a cell viability test on L929 cells (Figure S2 and Note S1, Supporting Information). As the flow drag is highly related to the shape of the object and a streamlined body can remarkably reduce the resistance owing to a lower pressure differential across its cross‐sectional area, we designed the robot to have a spindle structure with a fineness ratio (ratio of maximum length to maximum thickness) of ≈4.

A cylindrical magnet (N38, diametrically magnetized, 1.5 mm × 5 mm) was placed at the center of the robot as the remote engine. When a gradient magnetic field is applied, the robot presses close to the vessel wall, where the subject movement resistance can be maximumly reduced owing to the low laminar flow speed at the boundary. Furthermore, when a rotating gradient magnetic field revolves and moves forward along the blood vessel, the robot can move forward at the same velocity in a spiral‐rolling manner (Figure 1a).

Inspired by the labellar structure of the fly's mouthparts, we cut the tail of the robot into two pieces (Figure 1b), inside which a fine needle (outer diameter 310 µm) was concealed for endovascular biopsy. During the locomotion of the robot, the tail can cover the needle, like a sheath, preventing it from contacting blood vessels. For biopsy purposes, we can easily deform the morphology of the tail and expose the needle to harvest the target tissue by applying a stronger gradient magnetic field (magnetic flux density ≈ 220 mT, magnetic field gradient ≈ 550 Gs cm^−1^) from the desired direction.

We designed a pressure‐based release strategy inspired by the digestive system of mosquitoes to realize the controllable delivery in the blood vessel (Figure 1c). A cavity (cylindrical hole, diameter 0.3 mm, length 5 mm) was cast on the opposite side of the needle, ≈0.2 mm away from the magnet and ≈0.3 mm away from the bottom surface of the robot (Figure S3, Supporting Information). At the end of the cavity, an elastic crack (length of ≈300 µm) was torn as a self‐sealing orifice, which can remain closed at the natural condition and open when a certain amount of pressure is applied. Moreover, once the applied pressure is removed, the crack recovers to its original shape to seal the cavity benefiting from the elasticity of the silicone rubber.

### 2.2 Robot Driving and Locomotion

**Figure 2:**
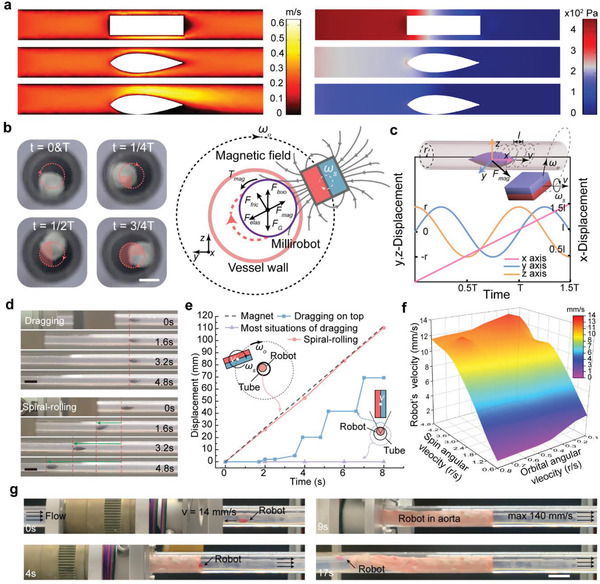
Robot driving and locomotion. a) Flow velocity and pressure simulation with a cylindrical design (top) and the robot at the center of the lumen (middle) or on the inner wall (bottom). FEA software (COMSOL Multiphysics 5.5) was used for the analysis where the vessel diameter is 5 mm and the input flow velocity is 0.2 m s^−1^. b) Cross‐section view of the rolling locomotion. The left four figures show the robot's position in one period when rolling in a plastic tube (scale bar 2.5 mm). c) Displacement of the robot rolling inside the lumen. The robot's trajectory will be a spiral when the external magnetic field has a constant velocity *v* on the *x*‐axis. d) In vitro dragging and spiral‐rolling test. In most dragging situations, the robot would not move. By spiral‐rolling locomotion (*ω*_s_ = 4.8 r s^−1^ and *ω*_o_ = 0.3 r s^−1^ separately, and the magnetic field gradient was ≈45 Gs cm^−1^), the robot was well controlled by the external magnet. Scale bars: 10 mm. e) Displacement time graph for dragging and spiral rolling. The external magnet moved forward at 14 mm s^−1^ in all the tests. During the locomotion for 8 s, only the robot with spiral‐rolling locomotion (*ω*_s_ = 4.8 r s^−1^ and *ω*_o_ = 0.3 r s^−1^ separately) was well controlled to follow the magnet. f) Measured robot velocity versus the spin and orbital angular velocities of the external magnetic field. The *z*‐axis indicates the robot's velocity in a 5 mm silicone tube upstream against a flow of 136 mm s^−1^ (mean flow rate ≈ 2680 mm^3^ s^−1^). g) Ex vivo locomotion experiment in the descending thoracic aorta of a sheep. The maximum flow velocity in the aorta was ≈140 mm s^−1^, and the robot's velocity was 14 mm s^−1^. Scale bar: 25 mm.

To demonstrate the superiority of the streamlined shape and on‐the‐wall motion in reducing the flow resistance, we conducted finite element analysis (FEA) in a tube with an inner diameter of 5 mm by providing a vena‐cava‐like flow condition (flow viscosity 4 mPa s and mean flow speed 20 cm s^−1^). The results suggest that the drag resistance of the robot (≈1.465 × 10^−3^ N) was reduced by ≈59.4% compared with that of the cylindrical shape (3.605 × 10^−3^ N). Moreover, as the lower‐velocity laminar flow near the interior wall yields a lower pressure differential, the strategy of on‐the‐wall motion can further reduce the movement resistance of the robot by 25.5% (1.091 × 10^−3^ N) compared with that at the center (**Figure** **2**a).

We propose a spiral‐rolling strategy to drive the robot to realize the forward locomotion along the blood vessel wall. Under the control of an external magnetic field, the robot revolves around the center of the vessel lumen and on its axis (Figure 2b; Note S2, Supporting Information), and finally exhibits a spiral trajectory if the magnet moves forward at a constant speed (Figure 2c). Compared with the direct dragging, which has been widely applied in the guidance of micro/nanorobots or catheters, the spiral‐rolling strategy demonstrates superior efficiency and controllability because it converts static friction into lower dynamic friction. As shown in Figures 2d,e, the spiral‐rolling strategy ensured that the robot moved forward uniformly for approximately 111 mm in 8 s following the magnet (red line in Figure 2e). In contrast, the robot always became stuck when driven by direct dragging. Although the direct dragging could move the robot in a special case where the magnet was located on top of the tube, the movement was discontinuous, and it was difficult to control the position of the robot (blue line in Figure 2e and Video S3, Supporting Information). A detailed mathematical model and analysis for the driving by spiral‐rolling and dragging can be found in the Supporting Information (Figures S4–S6, Notes S3 and S4, Supporting Information).

We quantitatively evaluated the upstream movement ability of our robot using in vitro and ex vivo experiments (Figure S7, Supporting Information). During the in vitro test, the robot was placed in a silicone tube (inner diameter of 5 mm) with a blood phantom (mean flow rate of ≈2680 mm^3^ s^−1^, mean flow velocity of ≈136 mm s^−1^), and a weak magnetic field (12 mT, 45 Gs cm^−1^) was applied for actuation. As shown in Figure 2f, the robot moved forward at a speed of 14 mm s^−1^ following the movement of the magnet along the tube. To test the moving ability of our robot in a real application, we conducted an ex vivo experiment in the descending thoracic aorta of a sheep with an irregular cross‐section (Figure 2g; Video S4, Supporting Information). Similar to the in vitro results, the robot could be well controlled at a stable speed of 14 mm s^−1^ to pass through the 18 cm aorta in less than 13 s. The results indicate that our robot can move upstream in a sophisticated environment through changes in the lumen diameter and flow rate, suggesting that the spiral‐rolling locomotion strategy is feasible and effective for in vivo applications.

### 2.3 Self‐Sealing Orifice for Controllable Drug Delivery

**Figure 3:**
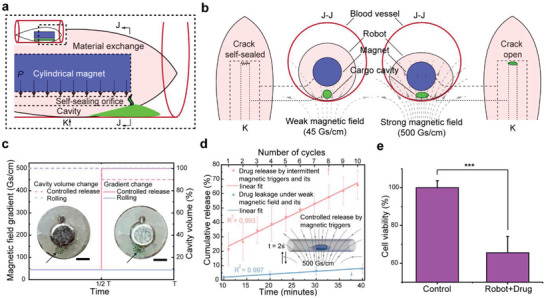
Self‐sealing orifice for controllable drug delivery. a) Illustration of pressure‐based release from the cavity. When a strong magnetic field (500 Gs cm^−1^) is provided, the cylindrical magnet will press the cavity and squeeze the liquid through the self‐sealing orifice. b) Status of the self‐sealing orifice when subjected to the weak (for locomotion) and strong (for drug release) magnetic field. c) Magnetic field and cavity volume change during releasing and rolling. The arrow points at the cross‐section of the cavity. The left figure shows the original round shape, and the right figure shows deformation to an oval shape under the strong magnetic field. Scale bars: 1 mm. d) Cumulative controlled release by 10 intermittent magnetic field triggers (500 Gs cm^−1^) and leakage test under a weak magnetic field (45 Gs cm^−1^ for driving the robot) for 40 min. e) Cell viability test on Hep‐G2 cells. The test group (Robot + Drug) was treated with doxorubicin‐loading robots, and the cell viability was 65.6% (*n* = 5, ***p < 0.001. Error bars indicate the SD).

Controllable drug delivery was realized using a pressure‐based release strategy through a self‐sealing orifice. As illustrated in **Figure** **3**a, when a magnetic field (200 mT, 500 Gs cm^−1^) was applied to the robot, the cavity was pressed, leading to a volume decrease as the cross‐section changed from round to oval. Consequently, the orifice (length 300 µm) opened with an increase in the inner pressure, and then squeezed out the material in the cavity (Figure 3b). Benefiting from the biocompatibility of the cavity and the physical release strategy, our robot is capable of carrying all materials that can dissolve or remain in liquids, such as drugs, particles, biologically active substances, liquid materials, and even cells (Figure S8, Supporting Information). Note that the magnetic field required for locomotion is ≈45 Gs cm^−1^, which is one order of magnitude smaller than the magnetic field gradient required to sustain the crack (Figure 3c). Hence, the orifice can remain sealed during motion and can recover to seal under the action of the natural elasticity of the silicone material once the strong magnetic field is removed after release.

The controllability of the delivery system was quantitatively evaluated using a methylene blue (MB) solution release test (Figure S9, Supporting Information). First, a syringe with a 30G needle (≈300µm) was used to inject the MB solution into the cavity through the self‐sealing orifice. The robot containing the MB solution (≈0.3 µL) in the cavity was placed in a tube, and then, a weak magnetic field (12 mT, 45 Gs cm^−1^) was applied to simulate the magnetic field applied in locomotion. The drug concentration in the tube was measured every 10 min for 40 min using a spectrometer, and the results indicated that the drug leakage was less than 10% (Figure 3d). On the other hand, when a stronger magnetic field of 500 Gs cm^−1^ was applied, ≈9.8% of the drug was released in 2 s (Figure S10, Supporting Information). We implemented intermittent release (2 s for each press) 10 times and observed that 65.8% of the drug was finally released (Figure 3d), which was consistent with the theoretical calculation (64.3%). This result showed the good controllability of the pressure‐based drug‐releasing strategy.

We performed a cytotoxicity test for the anticancer effect of doxorubicin (DOX) in Hep‐G2 cells to verify the drug delivery function of our robot further. Here, we released the drug (0.3 µL 0.55 mg mL^−1^ DOX) from the cavity by 10 intermittent magnetic triggers (2 s for each). The statistical data showed that the cell viability was reduced to 65.6% owing to the toxicity of DOX to Hep‐G2 cells (Figure 3e), suggesting the successful release of the drug. Moreover, we observed the cellular uptake result using fluorescence microscopy after incubation for 24 h. As shown in the fluorescence images (Figure S11, Supporting Information), DOX was internalized into almost all the nuclei of Hep‐G2 cells in the experimental group, further confirming the effectiveness of the drug delivery module.

### 2.4 Easy‐to‐Open and Auto‐Closed Tail for Biopsy

**Figure 4:**
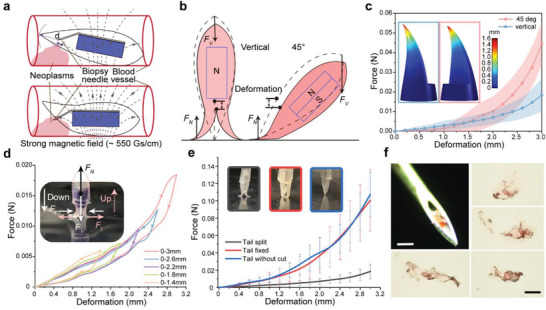
Easy‐to‐open and auto‐closed tail for biopsy. a) Mechanism of biopsy. During the rolling locomotion, the weak magnetic field will not expose the needle. For biopsy, the strong magnetic field will expose the needle to access the target tissue. b) Deformation of two typical tails (to expose the biopsy needle) by vertical or 45° contact. The dashed lines show the original position of the robot. c) Relationship between the vertical force or 45° force, and the deformation of the tails. The inner images are obtained via simulation using COMSOL to show a 1.6 mm deformation. d) Loading (down) and unloading (up) vertical force versus the deformation of the tails. This indicates that the tails have sufficient elasticity to close after the biopsy. e) Vertical force against the deformation changes of the tails for different structures. The “tail split” is our pre‐cut structure. The “tail fixed” is similar to the former, but only the tip of the two halves are not split. The “tail without cut” indicates no changes from the original structure. Our pre‐cut tail needs the lowest force for a 3 mm deformation. Error bars indicate the SD. f) Biopsy needle tip with a phantom tumor piece and the microscopic images (4×, Nikon) of collected samples. Scale bars: 300 µm.

Inspired by the structure of the fly's mouthparts, the EAT (easy‐to‐open and auto‐closed tail) biopsy system was proposed to realize an untethered percutaneous endovascular biopsy. Here, the biopsy needle was entirely concealed inside the two halves of the tail, with its tip ≈2.5 mm away from the tip of the robot. Such a design can hide the needle to prevent undesired scratches on the vessel wall during the motion of the robot. On the other hand, the needle tip can be exposed for biopsy by opening the two halves of the tail when a stronger magnetic field of ≈550 Gs cm^−1^ is properly applied (**Figure** **4**a; Figure S12, Supporting Information). As the exposure is only related to the distance between the needle tip and the robotic surface, a 3 mm deformation of the tail in the pre‐cut region is adequate regardless of the direction (Figure S13, Supporting Information). To illustrate this, we demonstrate two typical postures of the robot in biopsy in Figure 4b, where one is the ideal vertical contact (left), and the other is the side contact at an angle of 45° (right). As shown by the blue curve in Figure 4c, a force of 0.02 N could split the tail and expose the needle from the middle easily when the tail ideally contacted the target vertically. On the other hand, when the robot was at an angle to the target, a vertical force of ≈0.045 N could also bend the tail in the same direction and successfully expose the needle (red curve in Figure 4c). These results suggest that our biopsy method is robust in practice, regardless of the posture of the robot.

Moreover, owing to the natural elasticity of silicone, the tail can automatically close after a biopsy to protect the blood vessel wall from being scratched (Figure 4d; Note S5, Supporting Information). Furthermore, the pre‐cut tail in this study—that is, the 3 mm tail cut evenly in half from the middle of the plane—have a lower requirement for the applied force to expose the needle than a tail cut with other kinds of methods because the split tail requires a lower normal force to retain its deformation. As shown in the comparative results in Figure 4e, the split pre‐cut tail needs ≈0.02 N for a 3 mm deformation, whereas the other two structures, that is, the fixed tail and the tail without a cut, need ≈0.1 N for the same deformation, which is more than five times the former value.

To evaluate the effectiveness of the EAT biopsy system, we conducted an in vitro experiment in a 5 mm silicone tube, whose end was filled with a tumor phantom (Figure S14, Supporting Information). We applied a strong magnetic field (≈550 Gs cm^−1^) to the robot using a rectangular magnet for biopsy. Here, the needle was controlled to puncture the tumor phantom five times by moving the magnet close to and away from the tumor phantom (Figure S12a, Supporting Information). After retrieving the robot from the tube, we collected samples from the needle and observed them under a microscope (Figure 4f). Samples were successfully collected from the tumor phantom, indicating the capability of our robot for wireless controllable biopsy and its potential in clinical applications.

### 2.5 In Vivo Transportation, Delivery, and Biopsy Demonstration in Rabbit Aorta

**Figure 5:**
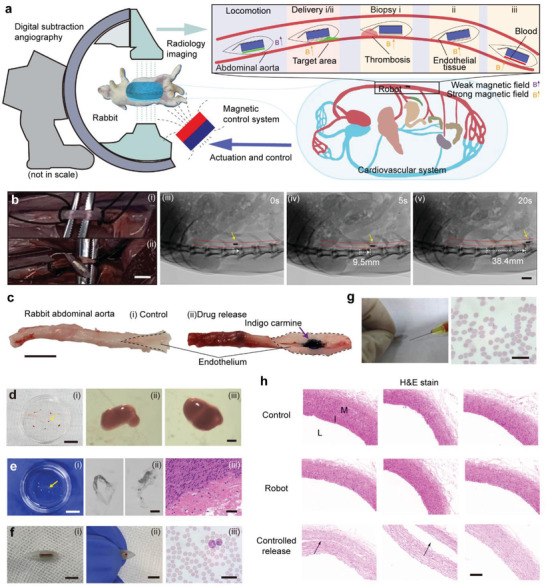
In vivo demonstration in the rabbit aorta model. a) Schematic of the biopsy and drug delivery in rabbit aorta. The magnetic field is applied for robot actuation and control, and medical imaging is used for position evaluation. During the locomotion in the aorta, only a weak magnetic field (45 Gs cm^−1^) is necessary. A strong magnetic field (500–550 Gs cm^−1^) is provided for biomedical applications. b) Rabbit abdominal aorta i) before and ii) after the experiment. iii–v) Medical images (DSA, Wandong) of our millirobot rolling in the aorta. Scale bars: 5 mm for (i) and (ii), 10 mm for (iii–v). c) In vivo controlled release of indigo carmine in a rabbit abdominal aorta (scale bar 10 mm). d) Biopsy of thrombosis and the microscopic images of the collected samples. Scale bars: 10 mm for (i), 200 µm for (ii) and (iii). e) Biopsy of the endothelium. Samples i) in a Petri dish, ii) under a microscope, and iii) Hematoxylin and Eosin (H&E) result. Scale bars: 10 mm for (i), 200 µm for (ii) and 50 µm for (iii). f) Liquid biopsy of blood. i) Blood sample in the cavity. ii) Squeezed blood from the cavity. iii) Blood samples under a microscope. Scale bars: 5 mm for (i), 3 mm for (ii) and 30 µm for (iii). g) Loading of red blood cells and image under a microscope. Scale bar: 25 µm. h) H&E results of the rabbit abdominal aorta. The sample for the robot group was collected after 10 min of locomotion. The sample for the controlled release group was collected after the delivery of hydrogen peroxide. The black arrows point at the vascular wall dissection caused by hydrogen peroxide. L, blood vessel lumen; I, tunica intima; M, tunica media. Scale bar: 100 µm.

We conducted an in vivo test in the rabbit abdominal aorta to demonstrate the capability of our millirobot for transportation, biopsy, and drug delivery further (**Figure** **5**a). As illustrated in Figure 5b, we inserted the robot into the rabbit's abdominal aorta, whose diameter (3.5–4 mm) was similar to that of the human coronary artery. The aorta was clamped during the subsequent in vivo experiments to prevent excessive blood loss. During the experiment, the position and posture of the robot were detected using digital subtraction angiography (DSA, CGO‐2100, Beijing Wandong Medical Equipment Co., Ltd.), which was used as feedback to control the magnetic field. As shown in the DSA images (iii–v) in Figure 5b, driven by a rotating magnetic field of ≈45 Gs cm^−1^, the robot could move forward for ≈40 mm in the blood vessel in 20 s and move back to its initial position within a similar time.

We filled the cavity with ≈0.3 µL blue stain (indigo carmine) to demonstrate the drug delivery ability in blood vessels. After driving the robot to the target position, a magnetic field of 500 Gs cm^−1^ was applied under the cavity for 2 s to press the cavity. This action was repeated ten times to release the blue stain to the vessel wall through the self‐sealing orifice. As shown in Figure 5c, approximately 13 mm^2^ of the vessel wall was stained with indigo carmine, indicating that our robot delivered the drug to the target region via a controlled release. Furthermore, a similar delivery process with hydrogen peroxide was performed. The optical image shows that the target area was corroded, and consequently became swollen and white (Figure S15, Supporting Information).

In vivo biopsy was also demonstrated considering blood clots and endothelial tissue as targets in the rabbit abdominal aorta. After the two halves of the tail were moved to the top of the blood clot, a strong magnetic field (550 Gs cm^−1^) was applied to expose the needle tip to contact and puncture the blood clot. An oscillating magnetic field was then applied to conduct the biopsy by shaking the magnet slightly (1 Hz). After repeating this action five times, a rotating weak magnetic field (45 Gs cm^−1^) was applied to conceal the needle and move the robot out of the aorta from the incision. Blood clot samples from the needle were collected for microscopic observation (Figure 5d). During the test, we successfully harvested six samples, and no endothelial tissue was collected. To demonstrate the controllability of the biopsy function further, we performed biopsy of the endothelium, which requires a more complex operation, because the needle cannot contact the endothelium when the robot is parallel to the blood vessel. Here, we rotated the strong magnetic field (500–550 Gs cm^−1^) by ≈30° to ensure that the head of the robot is up and its tail is down, followed by the parallel movement of the tilted magnetic field back and forth to scratch the vessel wall with the needle tip for biopsy. Approximately seven endothelium samples were collected (Figure 5e; Figure S16, Supporting Information), which could be used for studying the mechanism of vascular diseases and clinical decision‐making with further molecular analysis.^[^ ^28^, ^29^, ^30^, ^31^, ^32^ ^]^ Moreover, liquid biopsies (e.g., blood samples; Figure 5f) and live‐cell transportation (Figure 5g) can be conducted through the cavity, benefiting from biocompatible materials and pressure‐based release mechanisms.

Furthermore, the abdominal aorta was collected and observed after the in vivo experiment to evaluate how safe the robot is for the blood vessels during the operation. As shown in the histological images in Figure 5h, the H&E staining results indicated that the aorta structure (primarily the tunica intima) showed no difference compared with the control group. This indicates that the biopsy needle did not scratch the vessel wall, and the friction force did not harm the blood vessel during the rolling locomotion of the robot. On the other hand, after the controlled release of hydrogen peroxide, the aorta was corroded, which significantly differed from the control group. The black arrows in Figure 5h indicate the separation of the blood vessel wall. These comparative experiments confirmed the working ability, safety, and controllability of the robot in blood vessels.

## 3 Discussion

This paper presents a strategy for a multifunctional wireless millirobot that can swim upstream against fast blood flow, including controllable transportation, delivery, and endovascular biopsy, which may serve as a powerful platform for biomedical research and applications. For better upstream locomotion, a bioinspired streamlined shape and a strategy for spiral rolling on the interior wall of the blood vessel were proposed to reduce the flow resistance. The ex vivo and in vitro results (maximum flow velocity ≈ 140 mm s^−1^) demonstrated sufficient upstream spiral‐rolling ability in most blood vessels (mean blood flow velocity in the inferior and superior vena cava is 15 cm s^−1^, in the aorta is 40 cm s^−1^, and in capillaries is 0.03 cm s^−1^).^[^ ^33^ ^]^ For better performance in arteries with faster blood flow, the upstream movement could be further improved by the continued reduction of fluid resistance (e.g., the miniaturization of the robot) or by increasing the strength of the magnetic field. In this study, we designed a robot with a diameter ≈ 3 mm because this dimension is comparable to (or smaller than) that of several vascular interventional devices, such as atherectomy devices and ablation devices. Note that the robot can be further miniaturized using a smaller magnet (where a stronger magnetic field gradient for actuation and control would be necessary) and thinner silicone skin.

The integration of a self‐sealing orifice and elastic cavity provides a controllable wireless delivery method in blood vessels, which has been verified by DOX delivery to Hep‐G2 cells and drug release tests in the rabbit abdominal aorta. As the delivery is realized through a physical approach, this method is capable of carrying several types of materials if they can dissolve or remain in liquids. The capabilities of liquid (e.g., blood) biopsy and cell transportation provide opportunities for potential diagnostic and therapeutic applications. The vascular endothelium, a major blood–tissue interface, plays a critical role in maintaining vascular homeostasis. The gene expression analysis of vascular endothelium samples through endovascular biopsy could help researchers study the mechanism of cardiovascular diseases with the target vessels almost unchanged and may also aid in clinical decision making for future precise medicine. The representative EAT biopsy system is based on a pre‐cut tail inspired by the labellar structure of the fly's mouthparts, which can controllably collect samples from blood vessels and prevent the blood vessel from being scratched during robot movement. In this study, we used a normal commercial needle as a proof‐of‐concept. Note that the needle can be replaced with smaller or stimulus‐responsive devices (e.g., thermosensitive microgrippers) for higher efficiency and better safety. In addition, the biopsy tool can also be replaced by drug‐loading needles for vascular drug delivery,^[^ ^34^ ^]^ which may expand potential medical application scenarios.

Although limited by the current control system, the guidance of the robot in a winding pipe was demonstrated (Figure S17 and Video S5, Supporting Information), which preliminarily confirmed the feasibility of the spiral‐rolling strategy for winding blood vessels. In future work, we will attempt to design a control system with more degrees of freedom to promote precise guidance in real winding blood vessels. In addition, the robot can be further improved by integrating a sensor system, microfluidic system, or surgical tools to perform tests and surgeries on blood vessels. In addition to its demonstrated potential applications, our robot can also serve as a carrier to transport functionalized nanorobots that usually lack the ability to propel against the blood flow to the desired region. Moreover, the represented bioinspired structure could promote the development of untethered miniature devices for operation in natural lumens and cavities, which may contribute to a new era of wireless minimally invasive/non‐invasive inspections and surgeries.

## 4 Experimental Section

### Robot Fabrication

The soft millirobot was fabricated by microcasting (Figure S1, Supporting Information). Silicone rubber Ecoflex 00–30 (Smooth‐On, Inc.) part A and part B (1A:1B by weight) were mixed thoroughly for 3 min and put into a vacuum chamber to remove air bubbles. Then the mixture was poured into homemade 3D printing models (for flexible velamen) to cure at room temperature (23 °C) for 8 h before demolding. Inspired by the needle‐shaped labrum of the fly for feeding, a 30G needle (Zhejiang Kindly Medical Devices Co., Ltd.) was bent about 30° at the tip and attached to the center of the north pole of a diametrically polarized cylindrical NdFeB magnet (N38, 1.5 mm × 5 mm, Shenzhen Lala Magnet Material Co., Ltd.) using cyanoacrylate adhesive (Apollo 2240, Cyberbond L.L.C.). The assembly was then encased by a thin (200 µm) flexible velamen (Ecoflex 00–30, Smooth‐On, Inc.). The size of the hole was almost the same as the magnet. A steel wire (0.3 mm × 5 mm) was fixed to the center of the South pole of the magnet outside the flexible velamen via magnetic attraction. Next, the whole inner part was put into the hole of another set of homemade 3D printing models filled with silicone Dragon Skin 20 (Smooth‐On, Inc.). The liquid compound A and B (1A:1B by weight) of Dragon Skin 20 was prepared according to the manufacturer's instructions and cured at room temperature (23 °C) for 12 h. After demolding, the steel wire in the millirobot was removed from a small crack in the outer layer, which was made through a 30G fine needle. Then the millirobot was cut about 3 mm of the way through down the bottom lengthwise to expose the needle for biopsy. Finally, the robots were sterilized by autoclaving at 121 °C and 1.05 kg cm^−2^ (15–20 psi) for 20 min before in vivo experiment. For drug delivery demonstrations, a syringe with a 30G needle (≈300 µm) was used to inject the cargo (liquid) into the cavity through the self‐sealing orifice.

### Upstream Locomotion Test In Vitro and Ex Vivo

A soft silicone tube connected to a peristaltic pump that can provide a flow rate Q of ≈2680 mm^3^ s^−1^ was used to confirm the ability of the robot to move against flows. The inner diameter of the silicone tube was 5 mm, and the area A of the tube channel was *π* × (5/2)^2^ mm^2^. The average flow velocity *v* during the test was calculated by *Q*/*A*, and the result was ≈136 mm s^−1^. During the test, the robot was driven by an external magnet (45 × 45 × 20 mm; surface magnetization current density 5.637 × 105 A m^−2^; distance *d*_ML_ between the magnet center and lumen axis was 70 mm) to perform spiral‐rolling locomotion (spin angular velocity *ω*_s_ 0.6–4.8 r s^−1^, orbital angular velocity *ω*_o_ 0.1–0.8 r s^−1^), and it reached a velocity of 14 mm s^−1^ upstream. In the ex vivo experiment, the descending thoracic aorta (cross‐sectional area 38–113 mm^2^) of a sheep was connected to two peristaltic pumps to provide a flow of 5360 mm^3^ s^−1^. The flow velocity in the blood vessel varied from 48 to 140 mm s^−1^. The robot based on spiral‐rolling locomotion (*ω*_s_ = 4.8 r s^−1^, *ω*_o_ = 0.2 r s^−1^ and *d*_ML_ = 70 mm) successfully passed the 18 cm aorta at a velocity of 14 mm s^−1^.

### Controlled Release Experiment In Vitro

Methylene blue (MW 319.85, Aladdin) was used for leakage tests and controlled drug release. A syringe with a 30G needle (≈300µm) was used to inject the methylene blue (40 mg mL^−1^ solution prepared in DI water) into the cavity through the self‐sealing orifice. The release experiments were performed in centrifuge tubes (15 mL, LabServe, Thermal Fisher Scientific) that contained 4 mL DI water (resistivity ≈ 18 MΩ∙cm). For the leakage test, the millirobot was placed into a centrifuge tube for 10 min and moved to the next one for another 10 min under a weak magnetic field (12 mT, 45 Gs cm^−1^). For controlled release, the millirobot was placed in a centrifuge tube. An intense magnetic field (200 mT, 500 Gs cm^−1^) was then provided for 2 s using an external magnet to trigger the controlled release. After that, the millirobot was moved to the next tube to repeat the operation. The drug concentration was measured by a spectrophotometer at 664.9 nm, and then the amount of release could be obtained.

### Tumor and Blood Phantom Preparation

The tumor phantom was made by a gelatin‐agarose mixture to simulate the mechanical characteristics of a tumor. 1.412 g agar (D11291, OKA) and 2.212 g gelatin (240 g Bloom, Aladdin) were put into two centrifuge tubes (45 mL, LabServe, Thermal Fisher Scientific), respectively. The two centrifuge tubes were filled with 14.588 mL and 21.788 mL DI water (resistivity ≈ 18 MΩ∙cm) separately and then put into a 90 °C water bath. Waited until the two solutions have clarified, mixed the two materials in a new 45 mL centrifuge tube. When the solution was cooled to 36 °C, 0.38 mL 10% Formalin was mixed into it. Finally, the mixture was ready to be poured into a tube or other molds for congealing for 24 h at room temperature (23 °C). The blood phantom (blood‐mimicking fluid) was the mixture base of (% weight): DI water (85.41%); Glycerol (10.25%; Aladdin); Dextran from *Leuconostoc mesenteroides* (3.42%; D4876, Sigma‐Aldrich); and Dehypon LS 45 surfactant (0.92%; BASF).

### In Vitro Cytotoxicity

The robots were first put into a sterile glass bottle for standard sterilization (121 °C, autoclave for 30 min). After that, two robots were put into a 0.75 mL extraction medium (MEM culture medium with 10% Fetal Bovine Serum, Beyotime). The extraction medium was shaken for 24 h (37 °C, 100 rpm). Then the robots were removed, and the remaining solution was the extract of the sample. The positive control group was made by the extract of latex gloves (18 cm^2^) in a 3 mL extraction medium. The negative control group was the extract of 0.6 g high‐density polyethylene in a 3 mL extraction medium. The blank control was a 3 mL extraction medium. All extracts were prepared under the same conditions.

MTT assay was used to determine the cytotoxicity of our robot. L929 cells (Shenzhen Advanced Medical Services Co., Ltd.) were incubated in standard conditions before 0.25% trypsin digestion. The cells were diluted to 1 × 10^5^ cells per mL, plated into 96‐well plates (100 mL per well) and incubated for 24 h. Then the culture medium was removed from the wells, and 100 mL of different extracts (sample, negative control, positive control, and blank group) were added to each well (6 wells per group). The cells were incubated in standard conditions for another 24 h. After removing extracts, 50 mL MTT (1 mg mL^−1^) was added to each well and kept dark for 2 h. Then, MTT was replaced with 100 mL per well of isopropanol, followed by 10 min shake. The absorbance at 570 nm was measured according to the manufacturer's protocol. Cell viability was calculated by the following equation:

(1) $$\begin{array}{c} \mathrm{Cell}\;\mathrm{Viability}\;\left(\%\right)=\left(OD_{\mathrm{sample}}-OD_{\mathrm{blank}}\right)/\left(OD_{\mathrm{control}}-OD_{\mathrm{blank}}\right)\times 100 \end{array}$$

The OD_sample_ and OD_control_ were the absorbance values of the treated cells (as indicated) and the untreated control cells, respectively. The OD_blank_ was the absorbance of MTT itself at 570 nm.

The same L929 cells were also plated into a 24‐well plate (5 × 10^4^ cells per well) for Hoechst 33432 (C1026, Beyotime) staining. After standard incubation for 24 h, the culture medium was removed and replaced with 500 mL of different extracts (negative control, positive control, and blank group) for another 24 h incubation. Then the extracts were removed, and the cells were fixed with 0.5 mL 4% glutaraldehyde (Labcoms Life Sciences) for 20 min. After removing the fixing agent, the cells were washed with phosphate buffered saline (PBS) three times. Finally, the cells were stained with Hoechst 33342 for 5 min and washed with PBS three times. Samples were examined by fluorescence microscope (DM IL LED, Leica).

### Drug Delivery to Hep‐G2 Cancer Cells

CCK‐8 assay (KGA317, KeyGEN BioTECH) was carried out to evaluate the antitumor activity of DOX (Aladdin) delivered by the robot. The human liver carcinoma Hep‐G2 cells (Beijing Biobw Biotechnology) were incubated in standard conditions before trypsin (T1300, Solarbio) digestion. Then the cells were seeded in a 96‐well plate with a density of 1 × 10^4^ cells per well (six multiple holes for each group) at 37 °C in a 5% CO_2_ atmosphere for 24 h. Next, the culture medium (complete DMEM with high glucose and 10% fetal bovine serum supplemented, KeyGEN BioTECH) was replaced with 100 uL same fresh culture medium for the control and experimental group. For the experimental group, a drug‐loaded robot (0.3 µL DOX with 0.55 mg mL^−1^ concentration) was placed into each well for controlled release. An external magnetic field (200 mT, 500 Gs cm^−1^) was provided to trigger the drug release ten times, and then the robot was removed. The cells in the control and experimental group were cultured for another 24 h in the same condition. After the cells were washed three times with PBS, the CCK‐8 assay was used to detect the cell survival rate. 10 mL CCK8 was added to each well for 2 h, and the absorbance at 450 nm was measured according to the manufacturer's protocol. Cell viability was calculated by Equation (1) in Experimental Section.

For fluorescence microscopic observation, Hep‐G2 cells were seeded in 48‐well plates (2 × 10^4^ cell per well) at 37 °C in a 5% CO_2_ atmosphere for 24 h. The drug concentration was adjusted to 1.1 mg mL^−1^ to reach the same drug concentration in a 200 mL growth medium. Other conditions remained the same. After another incubation for 24 h at 37 °C, the supernatant was carefully removed, and the cells were washed three times with PBS. Subsequently, the cells were fixed with 4% formaldehyde (Labcoms Life Sciences) in each well for 15 min at room temperature and washed three times with PBS again. The nucleus of cells was dyed by Hoechst 33342 (C1026, Beyotime). Samples were examined by fluorescence microscope (CX53, OLYMPUS; blue fluorescence, nucleolus; red fluorescence, DOX).

### In Vivo Experiment

All in vivo experiments were performed on six New Zealand White male rabbits (aged 5–6 months, weighing 2.1–2.3 kg; purchased from Shenzhen Advanced Medical Services Co., Ltd.). The ethical approval from the Institutional Animal Care and Use Committee was obtained prior to the research. They were randomly divided into six groups, group 1 (for liquid biopsy and cell transportation), group 2 (for locomotion), group 3 (for indigo carmine delivery), group 4 (for hydrogen peroxide delivery), group 5 (for biopsy), and the control group. No animal was excluded from this study. The rabbits underwent 8 h of fasting treatment before the in vivo experiment. In brief, except for the control group, the rabbits were anesthetized, and the abdominal aortas were exposed through a laparotomy. A small incision less than 3 mm was made to deliver the millirobot into the aorta, followed by appropriate hemostatic measures. Then the aorta was clamped to prevent bleeding during the operations.

### Liquid Biopsy

For the liquid (blood) biopsy in the blood vessels, the cavity of the robot was filled with saline. The robot was sent into the aorta and moved forward about 3 cm (target area). The operation of liquid biopsy was the same as that of the pressure‐based release, in which the negative pressure would draw blood near the self‐sealing orifice when the cavity recovered to its original shape. Then the action was repeated ten times, and the robot was retrieved from the aorta. The blood sample in the cavity was squeezed on a glass side to make a whole blood smear. After being treated with Wright's stain, the sample was observed under a microscope.

### Safety Test for Cell Transportation

The red blood cells (RBCs) from the same rabbit were used to evaluate the safety of cell transportation. For preparing the RBCs, whole blood was taken from the rabbit and collected in a vacuum blood collection tube containing EDTA. The whole blood was then centrifuged at 800 × *g* for 20 min at 4 °C. The plasma on top was discarded, and the RBCs were collected at the bottom. Approximately 0.3 µL RBCs were injected into the cavity, and then the robot was delivered into the aorta for locomotion (10 min) using the rotating magnetic field (12 mT, 45 Gs cm^−1^). After that, the robot was taken out, and the RBCs in the cavity were squeezed on a glass side. Finally, a blood smear was made and stained with Wright's stain (C0135, Beyotime) for examination under a light microscope.

### Locomotion Test

A rotating external magnetic field (45 Gs cm^−1^) was provided to drive the robot in the rabbit's abdominal aorta. The position of the robot was detected by digital subtraction angiography (DSA, CGO‐2100, Beijing Wandong Medical Equipment Co., Ltd.). To avoid X‐ray exposure, magnetic actuation and imaging were conducted at intervals. The robot moved forward (to the distal end) for about 4 cm and moved backward at a similar time (20 s). Then the robot was retrieved from the incision. After that, the rabbits were euthanized, and the abdominal aortas were collected for histology analysis (H&E staining).

### Drug Delivery

Indigo carmine (PH9195, Phygene Scientific) and hydrogen peroxide (35% in H_2_O, Aladdin) were used to evaluate the efficiency of drug delivery. Approximately 0.3 µL indigo carmine solution (1 g mL^−1^ in DI water) or ≈0.3 mL hydrogen peroxide (used as received) was injected into the cavity of the robot. Before the delivery of the robot, the clamped abdominal aorta was flushed with normal saline 3–4 times to remove the blood for better observation. The robot moved toward the distal end for 2 cm (target area) by the rotating external magnetic field (45 Gs cm^−1^). A strong magnetic field (500 Gs cm^−1^ for 2 s, repeated ten times) was applied to press the cavity for drug release. Then the robot moved backward and was taken out from the incision. Both of the two animals were euthanized, and the abdominal aortas were collected for further observation. The aorta treated by hydrogen peroxide was further examined via histology analysis (H&E staining).

### Biopsy Test

For in vivo biopsy, blood clots and endothelial tissue were chosen as targets. Briefly, the thrombus was made through an injection of 0.5 mL fresh rabbit blood into a silicone tube (inner diameter 2 mm) and immersed in a 37 °C water bath for 3 h. The clot was stored at 5 °C for easy retraction. Next, the clot (recover to room temperature) was inserted into the blood vessel through the incision (≈3 cm from the incision). The distal end of the blood vessel was clamped to limit the movement of the clot. When the tail of the robot was on the thrombus, a strong magnetic field (550 Gs cm^−1^) was applied to expose the needle and contact the target. The external magnet was slightly shaken to make the needle tip puncture and cut the blood clot. After that, the robot was retrieved from the aorta. The clot samples were found at the surface or in the needle. Thrombosis samples were kept in PBS solution for further observation under a microscope. For endothelium biopsy, when the robot reached the targeted vessel, a strong magnetic field (500–550 Gs cm^−1^) was provided and then rotated about 30 deg to make the needle contact the tunica intima. The magnet was slightly moved to make the needle tip scratch the vessel wall and moved back. Such action was repeated five times before the robot was removed from the aorta. The endothelium samples from the needle were kept in PBS solution for further observation.

### Statistical Analysis

Sample size (*n* = 3) for each statistical analysis unless otherwise noted. Data were presented as mean ± SD. The statistical analysis was performed by IBM SPSS Statistics 26, followed by Student's *t*‐test and one‐way analysis of variance (ANOVA). *p < 0.05 was considered statistically significant. ***p < 0.001 was considered highly significant.

## Conflict of Interest

The authors declare no conflict of interest.

## Supporting information

Supporting Information

Supplemental Video 1

Supplemental Video 2

Supplemental Video 3

Supplemental Video 4

Supplemental Video 5

## Data Availability

The data that support the findings of this study are available in the supplementary material of this article.

## References

[1] M. Mimee , P. Nadeau , A. Hayward , S. Carim , S. Flanagan , L. Jerger , J. Collins , S. McDonnell , R. Swartwout , R. J. Citorik , V. Bulović , R. Langer , G. Traverso , A. P. Chandrakasan , T. K. Lu , Science 2018, 360, 915. 29798884 10.1126/science.aas9315 PMC6430580

[2] B. J. Nelson , I. K. Kaliakatsos , J. J. Abbott , Annu. Rev. Biomed. Eng. 2010, 12, 55. 20415589 10.1146/annurev-bioeng-010510-103409

[3] J. Li , B. E.‐F. de Ávila , W. Gao , L. Zhang , J. Wang , Sci. Rob. 2017, 2, eaam6431.

[4] L. Schwarz , M. Medina‐Sánchez , O. G. Schmidt , Appl. Phys. Rev. 2017, 4, 031301.

[5] V. Magdanz , I. S. M. Khalil , J. Simmchen , G. P. Furtado , S. Mohanty , J. Gebauer , H. Xu , A. Klingner , A. Aziz , M. Medina‐Sánchez , O. G. Schmidt , S. Misra , Sci. Adv. 2020, 6, eaba5855. 32923590 10.1126/sciadv.aba5855 PMC7450605

[6] H. Zhang , Z. Li , C. Gao , X. Fan , Y. Pang , T. Li , Z. Wu , H. Xie , Q. He , Sci. Rob. 2021, 6, 9519eaaz. 10.1126/scirobotics.aaz9519 34043546

[7] Z. Ren , R. Zhang , R. H. Soon , Z. Liu , W. Hu , P. R. Onck , M. Sitti , Sci. Adv. 2021, 7, p.eabh2022. 10.1126/sciadv.abh2022 PMC8245043 34193416

[8] A. Abramson , E. Caffarel‐Salvador , M. Khang , D. Dellal , D. Silverstein , Y. Gao , M. R. Frederiksen , A. Vegge , F. Hubálek , J. J. Water , A. V. Friderichsen , J. Fels , R. K. Kirk , C. Cleveland , J. Collins , S. Tamang , A. Hayward , T. Landh , S. T. Buckley , N. Roxhed , U. Rahbek , R. Langer , G. Traverso , Science 2019, 363, 611. 30733413 10.1126/science.aau2277 PMC6430586

[9] S. Jeon , S. Kim , S. Ha , S. Lee , E. Kim , S. Y. Kim , S. H. Park , J. H. Jeon , S. W. Kim , C. Moon , B. J. Nelson , J.‐y. Kim , S.‐W. Yu , H. Choi , Sci. Rob. 2019, 4, eaav4317.

[10] L. Ricotti , B. Trimmer , A. W. Feinberg , R. Raman , K. K. Parker , R. Bashir , M. Sitti , S. Martel , P. Dario , A. Menciassi , Sci. Rob. 2017, 2, eaaq0495. 10.1126/scirobotics.aaq0495 33157905

[11] X. Yang , W. Shang , H. Lu , Y. Liu , L. Yang , R. Tan , X. Wu , Y. Shen , Sci. Rob. 2020, 5, eabc8191. 10.1126/scirobotics.abc8191 33208522

[12] Z. Wu , L. Li , Y. Yang , P. Hu , Y. Li , S.‐Y. Yang , L. V. Wang , W. Gao , Sci. Rob. 2019, 4, eaax0613. 10.1126/scirobotics.aax0613 PMC7337196 32632399

[13] A. V. Singh , T. Jahnke , S. Wang , Y. Xiao , Y. Alapan , S. Kharratian , M. C. Onbasli , K. Kozielski , H. David , G. Richter , J. Bill , P. Laux , A. Luch , M. Sitti , ACS Appl. Nano Mater. 2018, 1, 6205.

[14] B. Wang , K. F. Chan , J. Yu , Q. Wang , L. Yang , P. W. Y. Chiu , L. Zhang , Adv. Funct. Mater. 2018, 28, 1705701.

[15] G. Chatzipirpiridis , O. Ergeneman , J. Pokki , F. Ullrich , S. Fusco , J. A. Ortega , K. M. Sivaraman , B. J. Nelson , S. Pane , Adv. Healthcare Mater. 2015, 4, 209. 10.1002/adhm.201400256 24986087

[16] Z. Wu , J. Troll , H.‐H. Jeong , Q. Wei , M. Stang , F. Ziemssen , Z. Wang , M. Dong , S. Schnichels , T. Qiu , P. Fischer , Sci. Adv. 2018, 4, eaat4388. 30406201 10.1126/sciadv.aat4388 PMC6214640

[17] S. Yim , E. Gultepe , D. H. Gracias , M. Sitti , IEEE Trans. Biomed. Eng. 2014, 61, 513. 24108454 10.1109/TBME.2013.2283369 PMC4023810

[18] D. Son , H. Gilbert , M. Sitti , Soft Rob. 2020, 7, 10. 10.1089/soro.2018.0171 31418640

[19] M. Sitti , Nat. Rev. Mater. 2018, 3, 74.

[20] Q. Wang , K. F. Chan , K. Schweizer , X. Du , D. Jin , S. C. H. Yu , B. J. Nelson , L. Zhang , Sci. Adv. 2021, 7, eabe5914. 33637532 10.1126/sciadv.abe5914 PMC7909881

[21] Y. Alapan , U. Bozuyuk , P. Erkoc , A. C. Karacakol , M. Sitti , Sci. Rob. 2020, 5, eaba5726. 10.1126/scirobotics.aba5726 33022624

[22] D. Ahmed , A. Sukhov , D. Hauri , D. Rodrigue , G. Maranta , J. Harting , B. J. Nelson , Nat. Mach. Intell. 2021, 3, 116. 34258513 10.1038/s42256-020-00275-x PMC7611213

[23] W. M. Sherk , M. S. Khaja , B. S. Majdalany , W. E. Saad , A. M. Udager , K. J. Cooper , D. M. Williams , J. Vasc. Interv. Radiol. 2019, 30, 54. 30409475 10.1016/j.jvir.2018.08.002

[24] N. J. Morrissey , J. Goldman , J. T. Fallon , P. L. Faries , M. L. Marin , L. H. Hollier , J. Endovasc. Ther. 2003, 10, 136. 12751945 10.1177/152660280301000126

[25] T. Quan , X. Li , H. Xu , Y. Lin , C. Liu , D. Li , S. Guan , J. Neurosurg. 2018, 131, 462. 30141756 10.3171/2018.3.JNS173143

[26] C. R. Narins , E. H. Howell , V. Krishnamoorthy , JACC Cardiovasc. Interv. 2018, 11, 2339. 30391384 10.1016/j.jcin.2018.06.012

[27] F. Soto , J. Wang , R. Ahmed , U. Demirci , Adv. Sci. 2020, 7, 2002203. 10.1002/advs.202002203 PMC7610261 33173743

[28] Z. Varga , A. J. Flammer , P. Steiger , M. Haberecker , R. Andermatt , A. S. Zinkernagel , M. R. Mehra , R. A. Schuepbach , F. Ruschitzka , H. Moch , Lancet 2020, 395, 1417. 32325026 10.1016/S0140-6736(20)30937-5 PMC7172722

[29] D. L. Cooke , D. B. McCoy , V. V. Halbach , S. W. Hetts , M. R. Amans , C. F. Dowd , R. T. Higashida , D. Lawson , J. Nelson , C.‐Y. Wang , H. Kim , Z. Werb , C. McCulloch , T. Hashimoto , H. Su , Z. Sun , Transl. Stroke Res. 2018, 9, 20. 28900857 10.1007/s12975-017-0560-4 PMC6040584

[30] L. Feng , D. M. Stern , J. Pile‐Spellman , Radiology 1999, 212, 655. 10478228 10.1148/radiology.212.3.r99au28655

[31] Z. Sun , H. Su , B. Long , E. Sinclair , S. W. Hetts , R. T. Higashida , C. F. Dowd , V. V. Halbach , D. L. Cooke , J. Biotechnol. 2014, 192, 34. 25450638 10.1016/j.jbiotec.2014.07.434 PMC4654108

[32] E. Winkler , D. McCoy , Z. Sun , D. Cooke , Stroke 2020, 51, A128.

[33] E. N. Marieb , K. Hoehn , Human Anatomy and Physiology, 9th ed., Pearson Education, Inc., New Jersey 2013.

[34] K. J. Lee , M. J. Goudie , P. Tebon , W. Sun , A. Khademhosseini , Adv. Drug. Delivery Rev. 2020, 165–166, 41. 10.1016/j.addr.2019.11.010 PMC7295684 31837356

