# Supplementary material for: Functionalized Spiral‐Rolling Millirobot for Upstream Swimming in Blood Vessel
Source: Adv Sci (Weinh). 2022 Mar 31;9(16):2200342. doi: 10.1002/advs.202200342 (PMC9165508; doi:10.1002/advs.202200342)
Supplement: Supplementary file 1 — Supporting Information [file ADVS-9-2200342-s001.pdf]

ADVANCED SCIENCE
Open Access

Supporting Information

for *Adv. Sci.*, DOI 10.1002/advs.202200342

Functionalized Spiral-Rolling Millirobot for Upstream Swimming in Blood Vessel

*Liu Yang, Tieshan Zhang, Rong Tan, Xiong Yang, Dong Guo, Yu Feng, Hao Ren, Yifeng Tang, Wanfeng Shang and Yajing Shen**

Supporting Information

Functionalized spiral-rolling millirobot for upstream swimming in blood vessel

*Liu Yang, Tieshan Zhang, Rong Tan, Xiong Yang, Dong Guo, Yu Feng, Hao Ren, Yifeng Tang, Wanfeng Shang, and Yajing Shen**

Supplementary Figures:

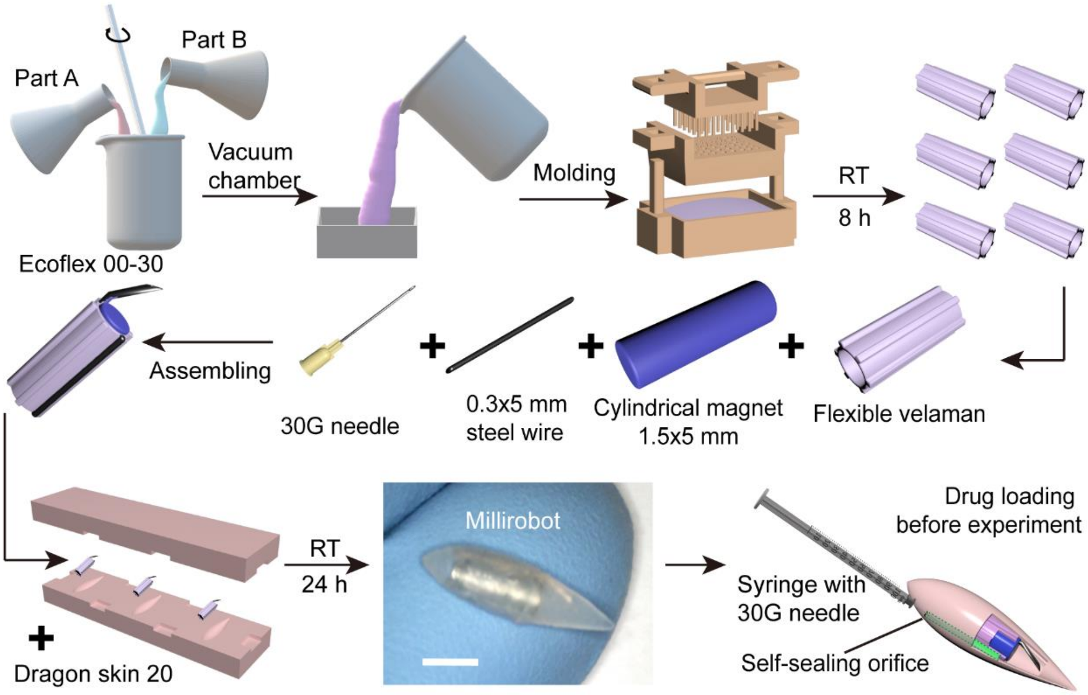

Figure S1. Fabrication steps. The soft millirobot was mainly fabricated by molding. Silicone rubber Ecoflex 00-30 (Smooth-On, Inc.) were mixed thoroughly and put into a vacuum chamber to remove air bubbles. Then the mixture was poured into homemade 3D printing models. A 30G needle was bent about 30 degrees at the tip and attached to the cylindrical magnet. The assembly was then encased by thin, flexible velamen. A steel wire was fixed to the center of the magnet's south pole outside the flexible velamen by magnetic attraction. Next, the whole inner part was put into the hole of another set of homemade 3D printing models filled with silicone Dragon Skin 20 (Smooth-On, Inc.). After demolding, the steel wire in the millirobot was removed from a small crack in the outer layer made by a 30G fine needle. A syringe with a 30G needle (~300μm) was used to inject the cargo (liquid) into the cavity through the self-sealing orifice. Scale bar: 3 mm.

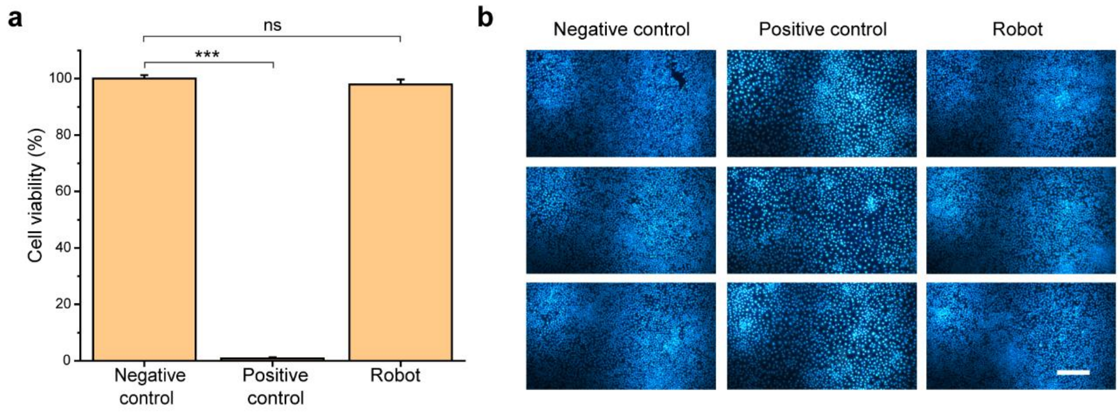

Figure S2. Cell viability test. a) Cell viability testing on L929 cells. No significance is found between the negative control group and the robot group. b) L929 cells with Hoechst 33342 staining (blue). The negative control group is treated with high-density polyethylene (no cytotoxicity), the positive control group is treated with latex gloves (highly cytotoxic), and the robot group is treated with the millirobot. The cells are observed by a fluorescence microscope. Error bars indicate SD. (n = 6, ***P < 0.001, ns means no statistically significant difference. Scale bar: 200 µm.)

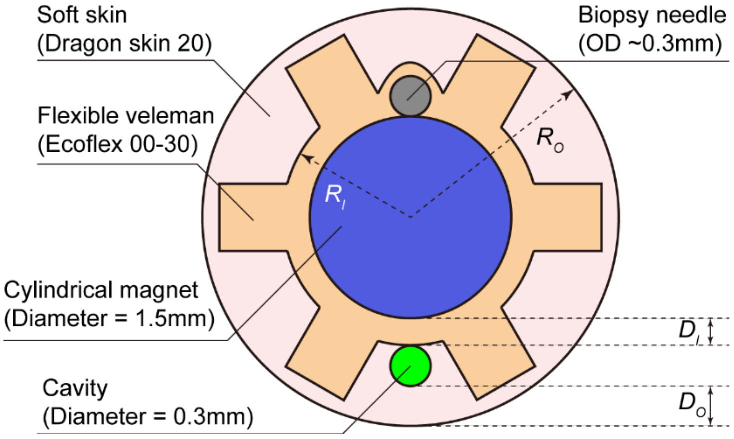

Figure S3. Cross-section view of the robot. The soft body of the robot is actually made of two silicones, Ecoflex 00-30 (Smooth-On, Inc.) and Dragon skin 20 (Smooth-On, Inc.). The distance between the magnet and the cavity is the thickness of the flexible velamen D_I = 200 μm. The distance from the cavity to the surface of the robot is D_O = 300 μm. The radius of the cross-section round R_O is about 1.55 mm, and R_I is ~0.95 mm.

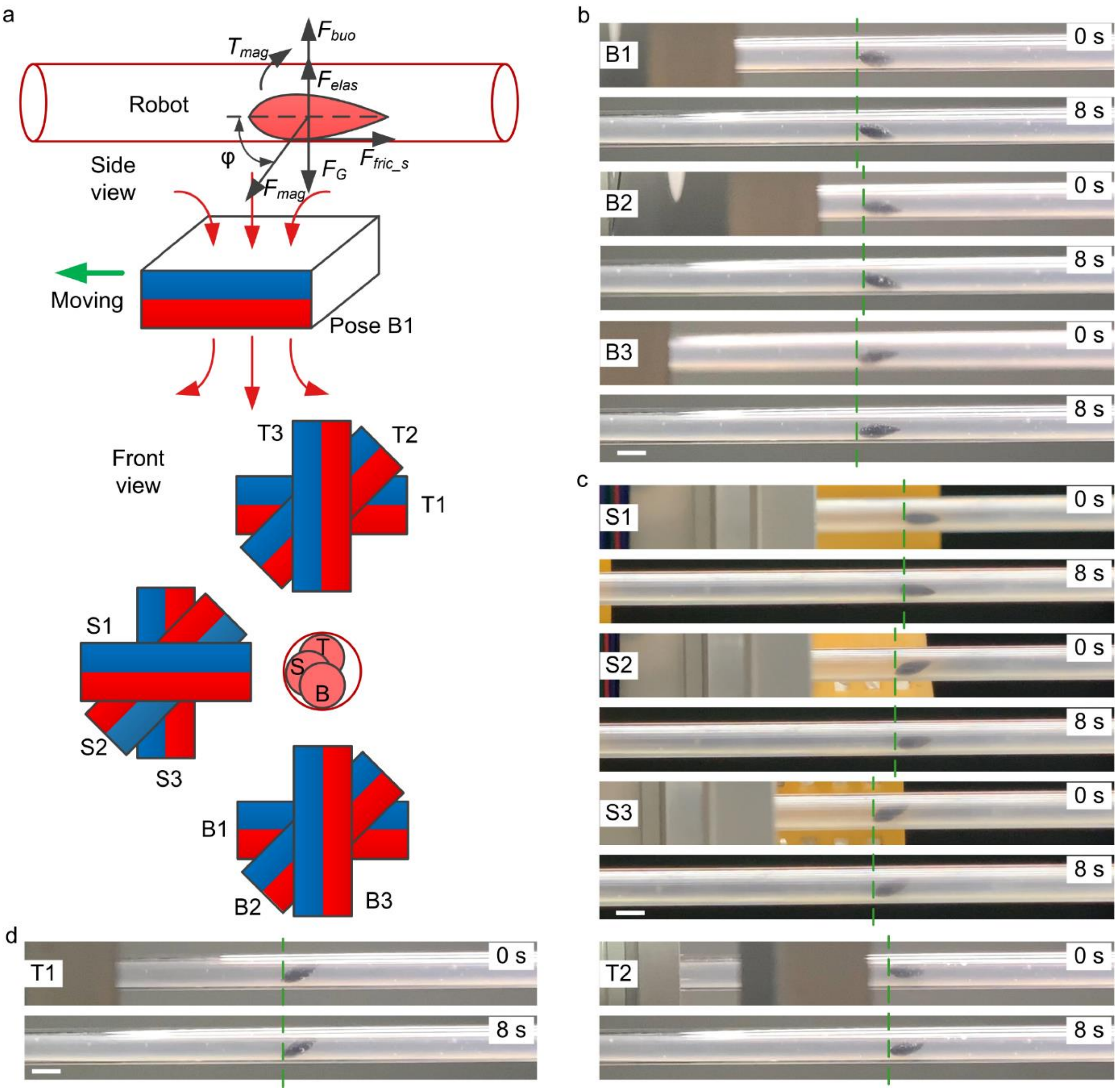

Figure S4. The direct dragging scheme under different actuation conditions. a) The schematic of magnetic actuation with different poses (B, S, and T denote bottom, side, and top position relative to the robot, respectively, while numbers 1, 2, 3 represent 0, 45, and 90 deg of the actuation magnet pose, respectively). The experimental process of the direct dragging scheme under bottom b), side c), and top d), position within an 8s period. The moving speed of the magnet is 14 mm s^{-1} for all the experiments. The green dotted lines denote the starting point of the robot. Scale bars: 5 mm.

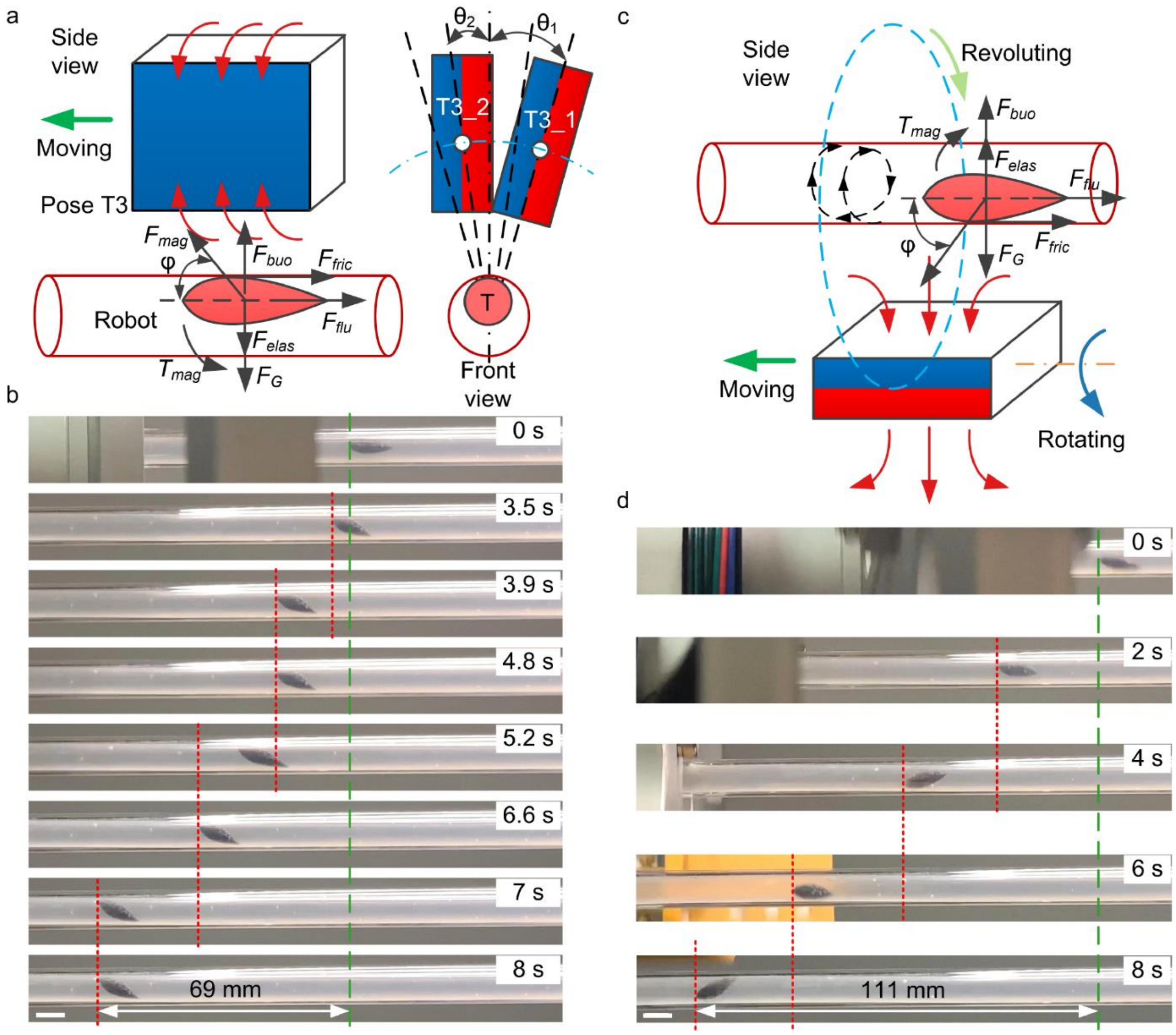

Figure S5. The actuation performance comparison between the direct dragging and the proposed spiral-rolling strategy. a) The schematic of the direct dragging actuation with a top position. There are angle limits θ_1 = 16° and θ_2 = 8° for the radial pose (T3_1) and the vertical pose (T3_2) to achieve an effective actuation, respectively. b) The experimental process of the actuation scheme T3 within an 8 s period. c)The schematic of spiral-rolling actuation strategy. d)The detailed process of the motion experiment under the spiral-rolling strategy during an 8 s period. The rotational and revolutionary speeds are 4.8 r s^{-1} and 0.3 r s^{-1}, respectively. The moving speed of the magnet is 14 mm s^{-1} for all the experiments. The green and red dotted lines, respectively, denote the starting point and instantaneous position of the robot. Scale bars: 5 mm.

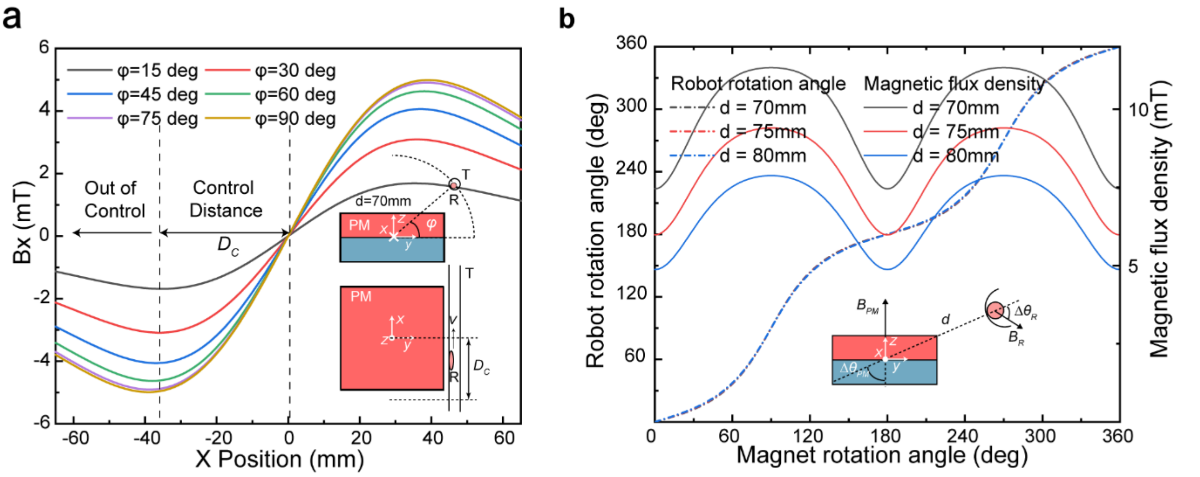

Figure S6. Simulation of the external magnetic field for spiral-rolling. a) Simulation results of the x-axial component (direction along the vessel) of the magnetic flux density for different rotation angles. Data were numerically calculated using MATLAB. (PM for permanent magnet, T for tube, and R for robot) b) Simulation results of the millirobot's rotation angle (dashed line) and magnetic flux density (solid line) versus the external magnet's rotation angle for different distances. Data were numerically calculated using MATLAB based on the model as follows:

Considering $P(x, y, z)$ is an arbitrary point outside a rectangular permanent magnet, which length, width and height denote as d_l, d_w and d_h, separately. The magnetic flux density $B(B_x, B_y, B_z)$ at P can be expressed as:

$$B_x = -(\mu_0 J/8\pi)[-T(x,y,z) - T(x,d_w - y,z) + T(d_l - x,y,z) + T(d_l - x,d_w - y,z)]|_{d_0=0}^{d_0=d_h} \quad (1)$$

$$B_y = -(\mu_0 J/8\pi)[-T(y,x,z) - T(y,d_l - x,z) + T(d_w - y,x,z) + T(d_w - y,d_l - x,z)]|_{d_0=0}^{d_0=d_h} \quad (2)$$

$$B_z = -(\mu_0 J/8\pi)[\Psi(x,y,z) + \Psi(y,x,z) + \Psi(d_l - x,y,z) + \Psi(d_w - y,x,z)$$

$$+\Psi(y,d_l - x,z) + \Psi(x,d_w - y,z) + \Psi(d_w - y,d_l - x,z) + \Psi(d_l - x,d_w - y,z)]|_{d_0=0}^{d_0=d_h} \quad (3)$$

where

$$T(\tau_1,\tau_2,\tau_3) = ln\frac{\sqrt{\tau_1{}^2+\tau_2{}^2+(\tau_2-d_0)^2}-\tau_2}{\sqrt{\tau_1{}^2+\tau_2{}^2+(\tau_2-d_0)^2}+\tau_2} \quad (4)$$

$$\Psi(\psi_1,\psi_2,\psi_3) = tan^{-1}[\frac{\psi_1(\psi_3-d_0)}{\psi_2\sqrt{\psi_1{}^2+\psi_2{}^2+(\psi_3-d_0)^2}}] \quad (5)$$

The permeability of vacuum μ_0 is $4\pi \times 10^{-7}$ H m^{-1}, J represents the surface density of the magnetizing current, and $[f]|_{d_0=0}^{d_0=d_h}$ represents the result of a function $[f]$ at $d_0 = d_h$ minus function $[f]$ at $d_0 = 0$.

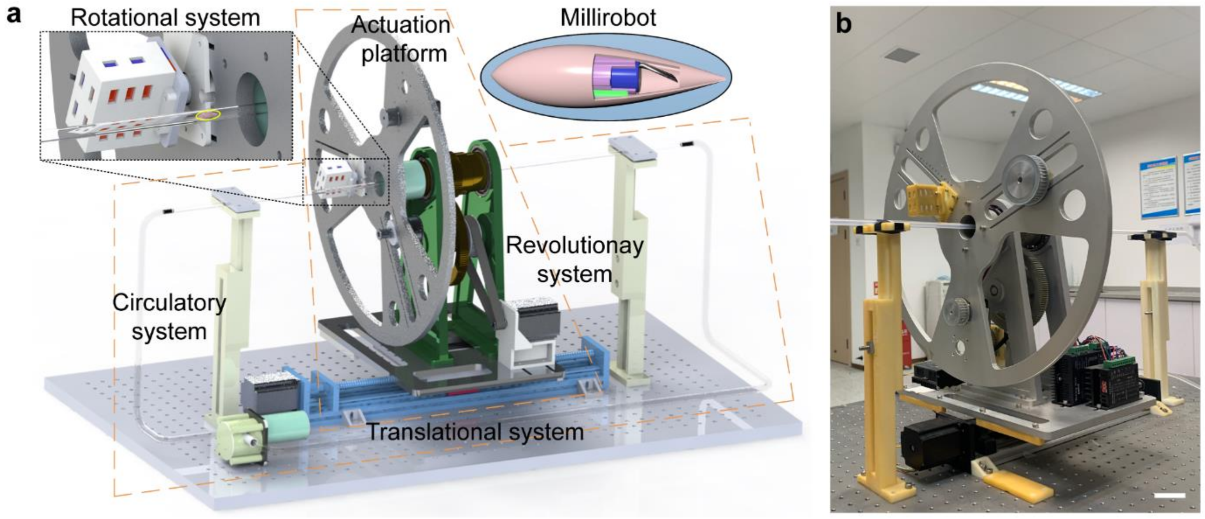

Figure S7. The experimental setup. a) The experimental setup mainly consists of three parts, including the actuation platform, the circulatory system, and the millirobot. There are three subsystems contained in the actuation platform, namely rotational subsystem, revolutionary subsystem, and translational subsystem. The circulatory system is set to simulate the intra-vascular dynamic fluidic environment. b) The picture of the control system. Scale bar: 30 mm.

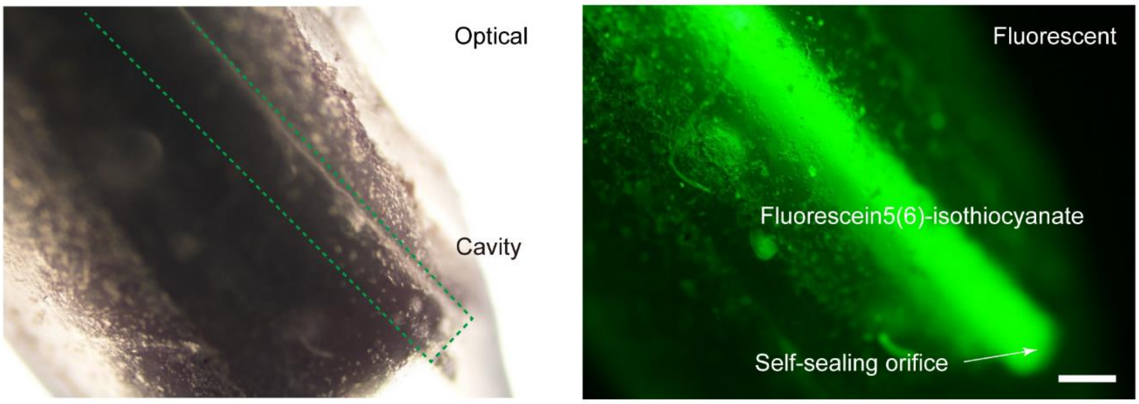

Figure S8. Optical and fluorescent images of the robot's cavity. The cavity was filled with fluorescent solution (Fluorescein5(6)-isothiocyanate, Aladdin). The cavity was well sealed, and the solution in it would not contact the cylindrical magnet. Therefore, the well-sealed biocompatible cavity can be used to carry different materials. Scale bar: 300 μm.

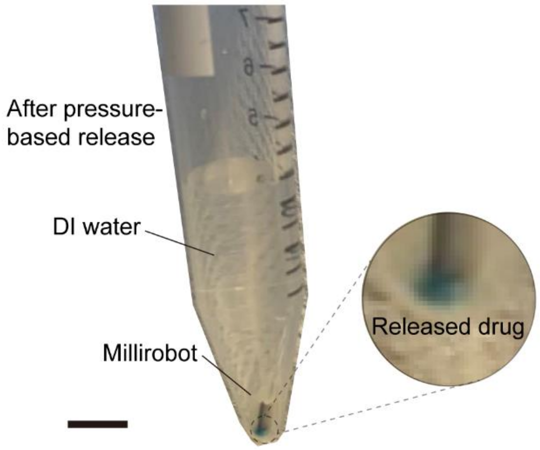

Figure S9. In vitro drug release (Methylene blue). This figure shows the pressure-based release of the drug in 4 mL water. After a strong magnetic field (200 mT, 500 Gs cm^{-1}) was applied for 2s, the robot was moved to another centrifuge tube with the same amount of water. The left water in the previous tube was sent to measure the drug concentration by a spectrometer, which was used to calculate the amount of drug release. For the leakage test, the measurement was the same. Scale bar: 10 mm.

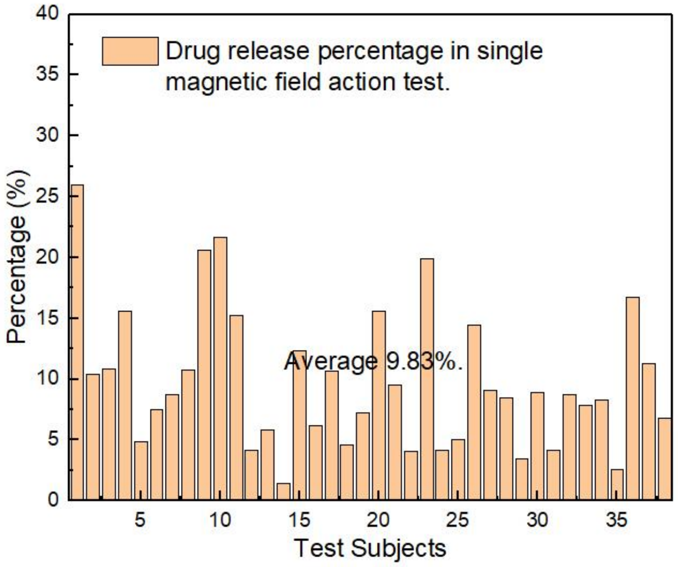

Figure S10. Percentage of drug release by single magnetic field trigger. For each test (details in **Experimental Section**), a strong magnetic field (500 Gs cm^{-1}) was provided for 2 s to trigger the pressure-based release. The average drug release percentage was 9.83%.

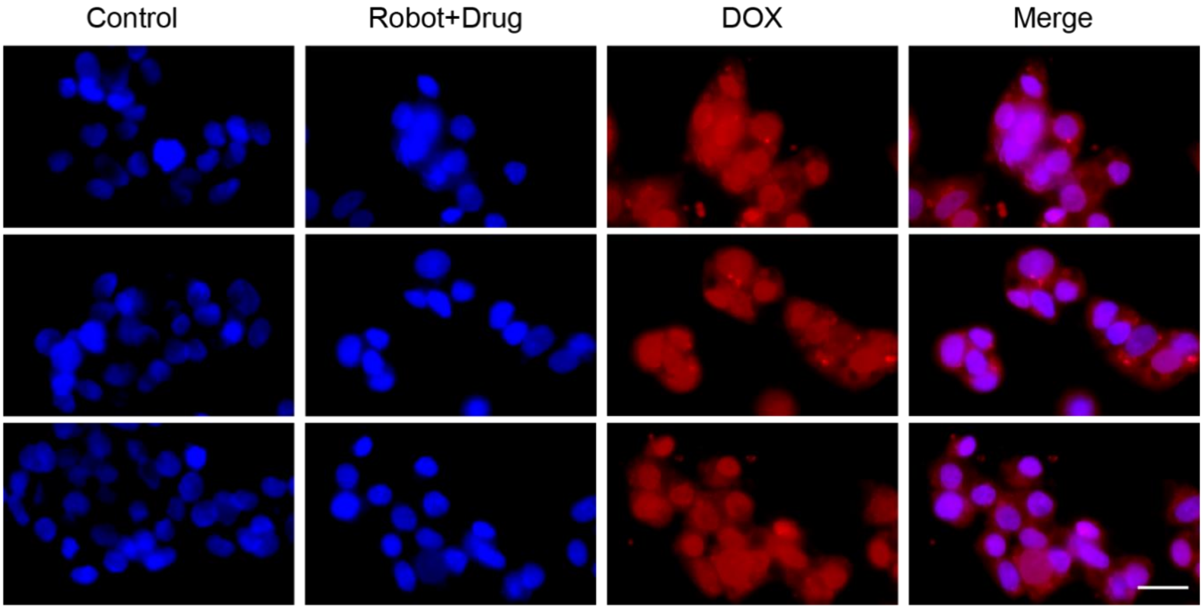

Figure S11. Hep-G2 cells with Hoechst 33342 staining. The fluorescence images showed the cellular uptake result after incubated with DOX released by our robot for 24 h. The blue fluorescence showed the nucleus of cells, and purple fluorescence (merge of nucleus and DOX) could be observed from almost all nuclei in the experiment group, which indicated that DOX was internalized into Hep-G2 cells effectively and further verified the effectiveness of the drug delivery module. (blue fluorescence, nucleolus; red fluorescence, DOX; purple, merge. Scale bar: 25 μm.)

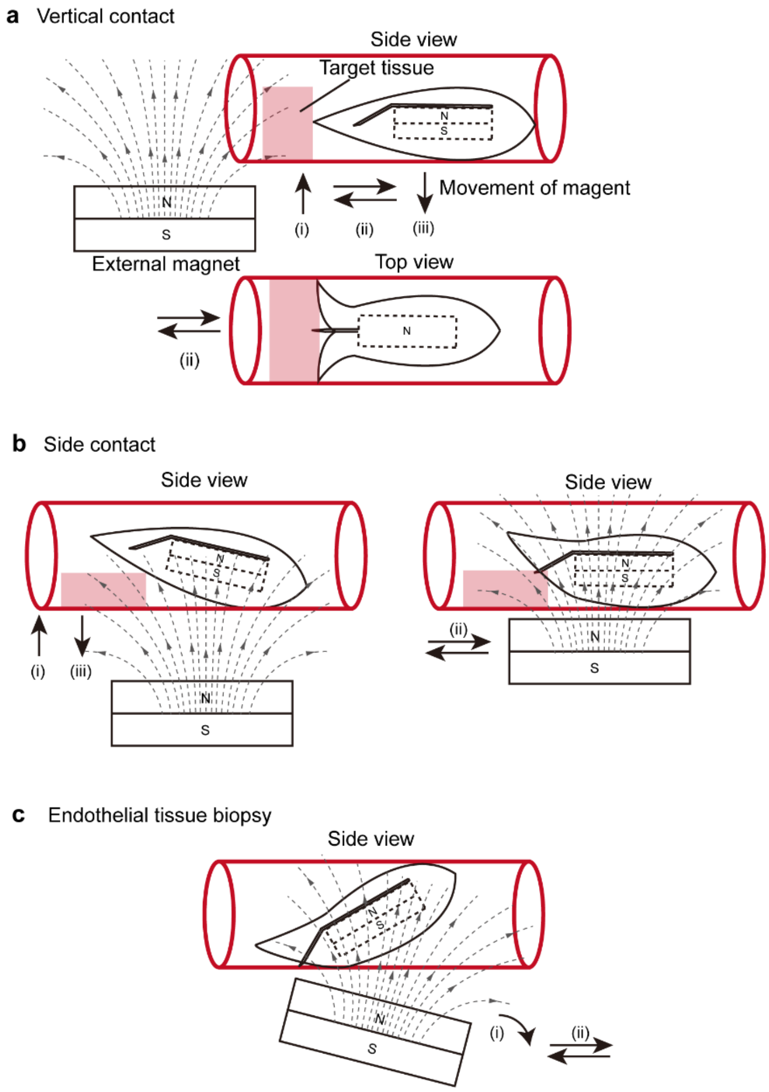

Figure S12. Methods to expose the needle for biopsy in different situations. For biopsy, the magnetic field gradient was about 550 Gs cm^{-1} for greater magnetic force and torque. a) When the target tissue is behind the robot, and the target basically blocks the blood vessel, we can use vertical contact. In short, it is to move the magnet closer to the robot and use the magnetic drag to open the tail of the robot, and the biopsy needle is pierced into the target tissue. Maintain the strong magnetic field and keep moving the magnet left and right to make the needle harvest the target tissue. Finally, the weak magnetic field is restored and used to drive the robot. b) When the target tissue is under the robot, we can use the method of side contact. Briefly, moving a magnet close to the robot provides a strong magnetic field and uses the attractive force of the magnet to press the tail of the robot against the target tissue. Move the magnet left and right to expose the biopsy needle and pierce the target tissue for sampling. c) To get the sample of tunica intima, we need an extra operation to make the needle contact the blood vessel wall. By rotating the magnet about 30 degrees, the robot's tail is moved down to contact the blood vessel wall.

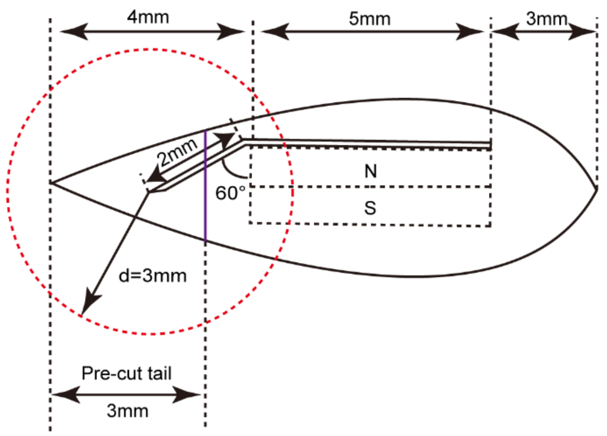

Figure S13. The distance from the needle tip to the surface of the robot. The length of the whole body is about 12 mm. The length of the magnet is 5 mm. The pre-cut tail is 3 mm. A 3 mm deformation is enough to expose the needle in the pre-cut tail's region.

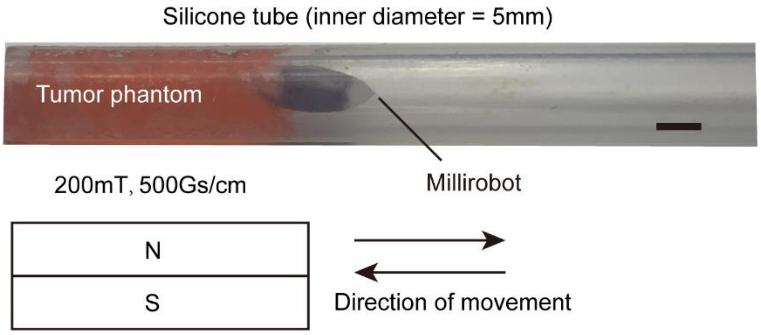

Figure S14. In vitro biopsy experiment in a tumor phantom. The in-vitro experiment was conducted in a 5 mm silicone tube, which end was filled with tumor phantom. A strong magnetic field (~200 mT, ~500 Gs cm^{-1}) was provided through a rectangular magnet for biopsy. The needle was controlled to puncture the tumor phantom about 5 times by moving the magnet close to and away from the tumor phantom. After retrieving the robot from the tube, we collected the samples from the needle. Scale bar: 3mm.

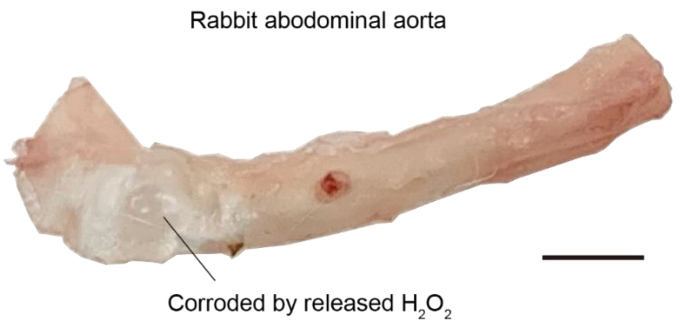

Figure S15. In vivo drug release (hydrogen peroxide) in the abdominal aorta of a rabbit. For evaluating the drug delivery efficiency, hydrogen peroxide was used. ~0.3 mL hydrogen peroxide was injected into the cavity of the Millirobot. A strong magnetic field (500 Gs cm^{-1} for 2 seconds, repeated 10 times) was applied to press the cavity for drug release. Then the robot moved backward and was taken out from the incision. The animal was euthanized, and the abdominal aorta was collected. The white region in the blood vessel wall was corroded by the released drug. Scale bar: 5 mm.

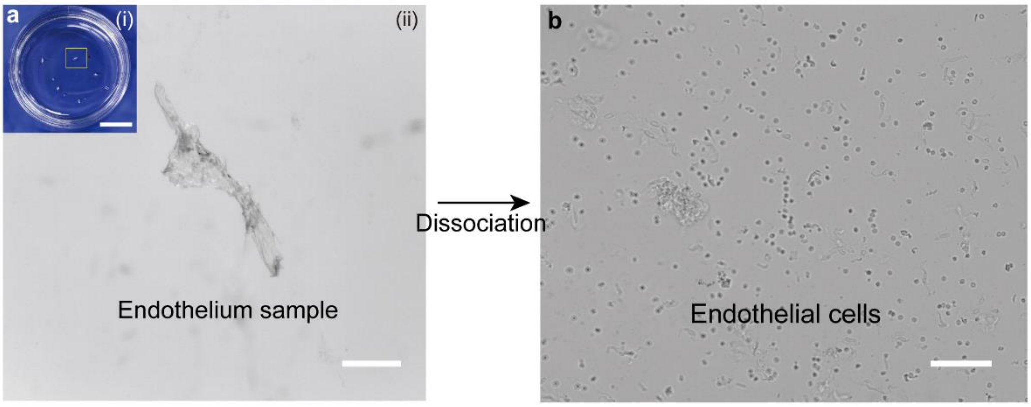

Figure S16. Rabbit abdominal aorta biopsy samples and endothelial cells. a) The biopsy samples of the endothelium in a Petri dish and one-piece under a microscope. Scale bars: 10mm for (i) and 200 µm for (ii). b) The endothelial cells were obtained from endothelium samples by EAT biopsy system. After the collection of samples from the needle tip, the samples were washed with PBS for 3 times and then put into endothelial cells dissociation buffer (0.5% bovine serum albumin, 2 mmol L^{-1} ethylenediaminetetraacetic acid, 0.1 mg mL^{-1} heparin in calcium- and magnesium-free PBS) for 15 min incubation at room temperature, followed by 12-15 rinses with above-mentioned dissociation buffer. Finally, the cells were centrifuged for observation. Noted that there may exist RBCs and other cells in the Petri dish, and they can be easily removed for special purposes (e.g., molecular analysis) through RBC lysing buffer and others. Scale bars: 100 µm.

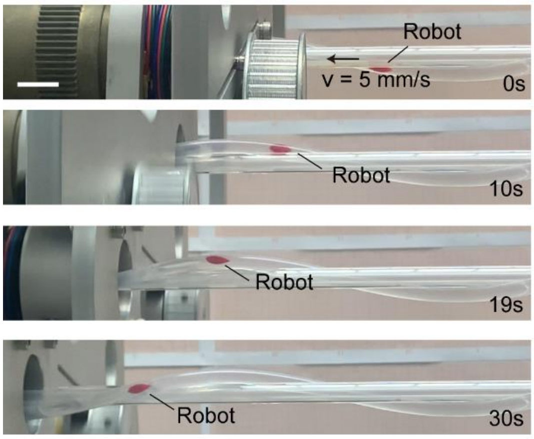

Figure S17. The guidance of the robot in a winding tube. The robot can move forward using the spiral-rolling strategy (spin angular velocity ω_s = 4.8 r s^{-1} and orbital angular velocity ω_o = 0.3 r s^{-1}) in a winding silicone tube with an inner diameter of 5 mm. Scale bar: 20 mm.

Supplementary Notes:

Supplementary Note S1. Cell viability test

A standard MTT test is introduced to evaluate the cytotoxicity of the millirobot. Figure S2 gives the cell viabilities of L929 cells cultured in the media treated with negative control, positive control, and our robot. The positive control causes significant cytotoxicity, while our robots do not show toxicity compared with the negative control group. The fluorescence images further verify the cytotoxicity test (Figure S2b). No significant difference is found between our robot and the negative control group, but the positive control group cells are less than the other two. These results indicate that our robot has good biocompatibility for potential biomedical applications.

Supplementary Note S2. Dynamics analysis for spiral rolling

This note describes the dynamics analysis of the robot during spiral rolling, as shown in Figures 2b and 2c. Here, in the y-z plane, both magnetic force F_{mag} and torque T_{mag} applied to the robot contribute to its locomotion, which can be described by:

$$F_{mag} + F_{buo} + F_{fric} + F_{elas} + F_G = m \cdot a \quad (1)$$

$$T_{mag} + T_{sum} = I \cdot \beta$$
(2)

where F_{buo}, F_{fric}, F_{elas}, F_G, m, a, T_{sum}, I, and β denote buoyancy, friction force, elastic force, gravity, mass, acceleration of circular motion, total torque other than from magnetic force, the moment of inertia and angular acceleration of rotation, separately. The centripetal acceleration $a = u^2 \cdot r^{-1}$ in a uniform circular motion (u, the linear velocity; *r*, the distance from the center of the robot to the center of the vessel) and angular acceleration β = 0 when the robot has a uniform rotation. The stable and sufficient F_{mag} and T_{mag} are the key to keep uniform circular motion and rotation on the vessel wall (in the *y-z* plane), in which the robot has the least flow drag.

As explained in Figure 2c, When the rotating external magnet moves forward at a constant velocity v, the increased distance between the magnet and the robot will create the component of the magnetic force in the x-axis direction, which is the power for the robot to achieve uniform motion with velocity v along the blood vessel eventually. Combined with the robot's circular motion in the plane perpendicular to the blood vessel and its uniform motion along the direction of the blood vessel, the robot's trajectory in the blood vessel is a spiral.

Supplementary Note S3. Analysis for dragging

To obtain the best locomotion performance, we evaluate two actuation schemes, including direct dragging and spiral rolling. Different actuation positions (bottom, side, top) and poses (0°, 45°, 90°) of

the direct dragging are first presented in Figure S4a. According to the experimental data, we know that there is no displacement occurs for the robot under the eight conditions (B1, B2, B3, S1, S2, S3, T1, and T2). Then the mechanical equilibrium of the robot (e.g., under the actuation pose B1) can be concluded as below.

$$F_{mag}\cos\varphi - F_{fric\_s} = 0 \quad (3)$$

$$F_{mag}\sin\varphi + F_G - F_{elas} - F_{buo} = 0 \quad (4)$$

$$T_{mag} - F_{fric\_s}R_O = 0 \quad (5)$$

$$F_{fric\_s} = \mu_s F_{elas} \quad (6)$$

where F_{mag}, T_{mag} , F_{fric_s}, F_G, F_{elas}, F_{buo}, μ_s, φ and R_O denote the magnetic force, magnetic torque, static friction force, gravity, elastic support force, buoyancy, static frictional coefficient, actuation angle and the robot's radius, respectively.

Once the actuation magnet moves forward, both F_{mag} and φ decrease while T_{mag} experiences an increase. Then F_{elas} and F_{fric_s} obtain a corresponding reduction. The robot possesses the tilted trend later. However, the actuation force along the tube is always smaller than the maximal static friction force, which means Equation (3) maintains equilibrium. The robot would not present displacement.

With the T3 actuation condition (Figure S5a), the robot can achieve an intermittent advancing motion (Figure S5b). Under this actuation condition, the maximal static friction force is slightly small and easy to be conquered by the actuation force along the tube. The intermittent motion means that the robot alternately experiences the sliding and static status. During the sliding process, the mechanical equilibrium of the robot changes into the following status.

$$F_{mag}\cos\varphi - F_{fric} - F_{flu} = 0 \quad (7)$$

$$F_{mag}\sin\varphi + F_{buo} - F_{elas} - F_G = 0 \quad (8)$$

$$T_{mag} - F_{fric}R_O = 0 \quad (9)$$

$$F_{fric} = \mu F_{elas} \quad (10)$$

where F_{flu}, F_{fric} and μ denote the fluidic resistance, sliding friction force and sliding frictional coefficient.

We further confirm that this intermittent motion can be achieved within a certain range of actuation angles. As shown in Figure S5a, the angle limit for the radial pose (T3_1) and the vertical pose (T3_2) are θ1= 16° and θ2= 8°, respectively. Obviously, intermittent motion is not controllable locomotion for the robot. However, given the spiral-rolling strategy (Figure S5c), the robot can achieve

continuous locomotion with a nearly uniform speed (Figure S5d). Once the robot breaks the equilibrium of static status, it maintains the controllable sliding process along the tube.

Supplementary Note S4. Analysis for magnetic flux density

The magnetic flux density along the vessel at different angles at a certain distance (70 mm) is calculated to ensure that the external magnetic field can control the spiral rolling locomotion. The results in Figure S6a show that the magnetic flux density increases from 0 to max at D_C and then decreases with distance from the center. The length of D_C has a small variation at different angles, but it is always greater than the half-length of the external magnet. It means if the flow rate was high enough to push the millirobot D_C away from the center, no more magnetic force could be provided, and the control would be lost. As shown in Figure S6b, the rotational angle of the robot is proportional to that of the external magnet. Due to the rotating motion of the external magnet and the robot, the magnetic attraction that keeps the robot rolling on the vessel wall is not constant. For a constant distance of 70 mm, the magnitude has a ~34% variation from 7.46 mT to 11.33 mT with angles. As the distance increase from 70 mm to 80 mm, the max magnetic flux density decreases to 7.87 mT, almost the same as the minimum value at 70 mm. Therefore, the factors of magnetic field distribution, such as distance and angle, should be accurately evaluated in the control of rolling locomotion.

Supplementary Note S5. Tails' elasticity test

To ensure the pre-cut tail can auto-close after the endovascular biopsy, we measured the loading (down) and unloading (up) vertical force versus the tails' deformation as a representative. The forces were measured by every 0.1 mm with different total deformations from 1.4 mm to 3 mm. As the vertical force curves in Figure 4d, they showed similar forces when we lifted up the robot (to simulate the process of auto close) compared with the loading forces, which means the tails could recover to their original shape without external forces. The hysteresis of the force curve was caused by the friction force during the down and up process.
